# Collagen COL22A1 maintains vascular stability and mutations in *COL22A1* are potentially associated with intracranial aneurysms

**DOI:** 10.1242/dmm.033654

**Published:** 2018-12-12

**Authors:** Quynh V. Ton, Daniel Leino, Sarah A. Mowery, Nina O. Bredemeier, Pascal J. Lafontant, Allison Lubert, Suman Gurung, Janice L. Farlow, Tatiana M. Foroud, Joseph Broderick, Saulius Sumanas

**Affiliations:** 1 Division of Developmental Biology, Cincinnati Children's Hospital Medical Center, Cincinnati, OH 45229, USA; 2 Division of Pathology, Cincinnati Children's Hospital Medical Center, Cincinnati, OH 45229, USA; 3 Department of Pediatrics, University of Cincinnati College of Medicine, Cincinnati, OH 45267, USA; 4 Department of Biology, DePauw University, Greencastle, IN 46135, USA; 5 Department of Medical and Molecular Genetics, Indiana University School of Medicine, Indianapolis, IN 46202, USA; 6 Department of Neurology and Rehabilitation Medicine, University of Cincinnati, Cincinnati, OH 45267, USA

**Keywords:** Intracranial aneurysms, Collagen, Zebrafish, Vascular integrity

## Abstract

Collagen XXII (COL22A1) is a quantitatively minor collagen, which belongs to the family of fibril-associated collagens with interrupted triple helices. Its biological function has been poorly understood. Here, we used a genome-editing approach to generate a loss-of-function mutant in zebrafish *col22a1*. Homozygous mutant adults exhibit increased incidence of intracranial hemorrhages, which become more prominent with age and after cardiovascular stress. Homozygous *col22a1* mutant embryos show higher sensitivity to cardiovascular stress and increased vascular permeability, resulting in a greater percentage of embryos with intracranial hemorrhages. Mutant embryos also exhibit dilations and irregular structure of cranial vessels. To test whether *COL22A1* is associated with vascular disease in humans, we analyzed data from a previous study that performed whole-exome sequencing of 45 individuals from seven families with intracranial aneurysms. The rs142175725 single-nucleotide polymorphism was identified, which segregated with the phenotype in all four affected individuals in one of the families, and affects a highly conserved E736 residue in COL22A1 protein, resulting in E736D substitution. Overexpression of human wild-type COL22A1, but not the E736D variant, partially rescued the *col22a1* loss-of-function mutant phenotype in zebrafish embryos. Our data further suggest that the E736D mutation interferes with COL22A1 protein secretion, potentially leading to endoplasmic reticulum stress. Altogether, these results argue that COL22A1 is required to maintain vascular integrity. These data further suggest that mutations in *COL22A1* could be one of the risk factors for intracranial aneurysms in humans.

## Abstract (precis)

**Summary:** Collagen COL22A1 is expressed in perivascular fibroblast-like cells and is required to maintain vascular stability in a zebrafish model. Mutations in COL22A1 are likely to be associated with intracranial aneurysms.

## INTRODUCTION

Collagens comprise a large family of extracellular matrix proteins, which play a key role in the tissue integrity of all organs. There are at least 28 distinct collagen types in humans. Fibrillar collagens are trimeric molecules composed of three polypeptide alpha chains, which contain the sequence repeat Gly-X-Y and form a triple helix (Ricard-Blum and Ruggiero, 2005). The major fibrillar collagens, such as collagen I, have a broad distribution and are present in multiple mesenchymal connective tissues, including bone, cartilage, tendons and others (Myllyharju and Kivirikko, 2001). The fibril-associated collagens with interrupted triple helices (FACIT) family of collagens comprise quantitatively minor collagens that often copolymerize into suprastructures with the major collagens and mediate ligand interactions between fibrils and their environment (Koch et al., 1995; Shaw and Olsen, 1991). FACIT proteins are involved in regulating the integrity and stability of the extracellular matrix (ECM) and its fibrillar collagen network (Ivanova and Krivchenko, 2014; Oudart et al., 2017). Collagen XXII (COL22A1) is one of the more recently identified collagens that belongs to the FACIT family. It contains an N-terminal von Willebrand factor-like domain, followed by a thrombospondin N-terminal domain and a long collagenous domain with several interruptions of Gly-X-Y repeats (Koch et al., 2004). COL22A1 is known to be concentrated at tissue junctions in the muscle, tendons, heart, articular cartilage and skin (Koch et al., 2004). It is deposited at the basement membrane of the myotendinous junction and was shown to be associated with the extrafibrillar matrix in cartilage (Koch et al., 2004). It has been reported that *col22a1* morpholino knockdown in zebrafish embryos disrupted the myotendinous junction and induced muscular dystrophy (Charvet et al., 2013). However, morpholinos are prone to off-target effects (Eisen and Smith, 2008; Kok et al., 2015). To date, no analysis of genetic mutants in Col22a1 has been reported in any vertebrate organism. Therefore, the biological role of the protein remains poorly understood.

In addition to playing the major role in structural integrity of the connective tissues, collagens are major components of the ECM within the wall of blood vessels. The blood vessel wall contains subendothelial basement membrane, intima, media, adventitia and interstitial matrix layers. Each of these layers contains multiple types of collagens, which are crucial for vascular stability and structural integrity. Collagen IV is a major component of the basement membrane, and mutations in human *COL4A1* have been associated with a variety of syndromes, including intracranial aneurysms and cerebral hemorrhages (Volonghi et al., 2010). Mutations in fibrillar collagens I, III and V have been associated with the Ehlers–Danlos syndrome and can result in arterial ruptures and aneurysms (Malfait, 2018). However, the role of FACIT collagens in maintaining vascular stability is currently poorly understood, and Col22a1 has not previously been implicated in vascular integrity.

Intracranial berry aneurysms (intracranial aneurysms; IAs) are small berry- or balloon-like defects in the wall of a major intracranial artery. Subarachnoid hemorrhage (SAH) accounts for ∼7% of stroke cases but has high rates of mortality (Butler et al., 2011). Most significantly, 30% of people who suffer from SAH die within a month (Feigin et al., 2003). SAH is caused by rupture of an IA, which can then damage the brain parenchyma. In addition to ruptured IAs, unruptured IAs, which leave individuals at an increased risk of SAH, are estimated to occur in 0.5-2.0% of the general population (Ingall et al., 2000). Currently, both genetic and environmental factors have been implicated in susceptibility to stroke.

Previous genome-wide association studies (GWAS) identified single-nucleotide polymorphisms (SNPs) in *EDNRA*, *SOX17*, *CDKN2BAS/ANRIL*, *CNNM2*, *KL/STARD13* and *RBBP8* associated with an increased risk for IAs (Bilguvar et al., 2008; Yasuno et al., 2011, 2010). To identify additional risk factors contributing to IA susceptibility, whole-exome sequencing (WES) was performed in seven families that had multiple affected individuals with IAs (Farlow et al., 2015). The data suggested susceptible candidate loci, although variants in *COL22A1* were not reported in the original study.

The zebrafish has emerged as an advantageous model system to study vascular development and function. Transparent embryos undergo external development and are easily accessible for experimental observations and manipulations. The molecular mechanisms that regulate vascular development and stability are highly conserved between multiple vertebrates, including zebrafish and humans. Therefore, zebrafish have been widely used to model and study multiple cardiovascular diseases in humans, including hemorrhagic stroke (Butler et al., 2011; Gore et al., 2012).

Here, we used a zebrafish model system to study the functional role of Col22a1. By utilizing zebrafish genetic mutants in *col22a1*, we show that Col22a1 plays an important role in promoting vascular stability. In addition, we reanalyzed the original data from the WES study of familial IAs (Farlow et al., 2015) and identified the rs142175725 SNP present in all affected individuals in one of the families, which is predicted to result in a E736D substitution within the COL22A1 protein sequence. We demonstrate that E736D perturbs COL22A1 function and results in hemorrhages when overexpressed in zebrafish embryos. Our results identify a novel role for Col22a1 in maintaining vascular stability and suggest that mutations in *COL22A1* could be one of the causes of intracranial aneurysms.

## RESULTS

### *col22a1* is expressed in perivascular fibroblast-like cells in zebrafish and mice

**Fig. 1.**
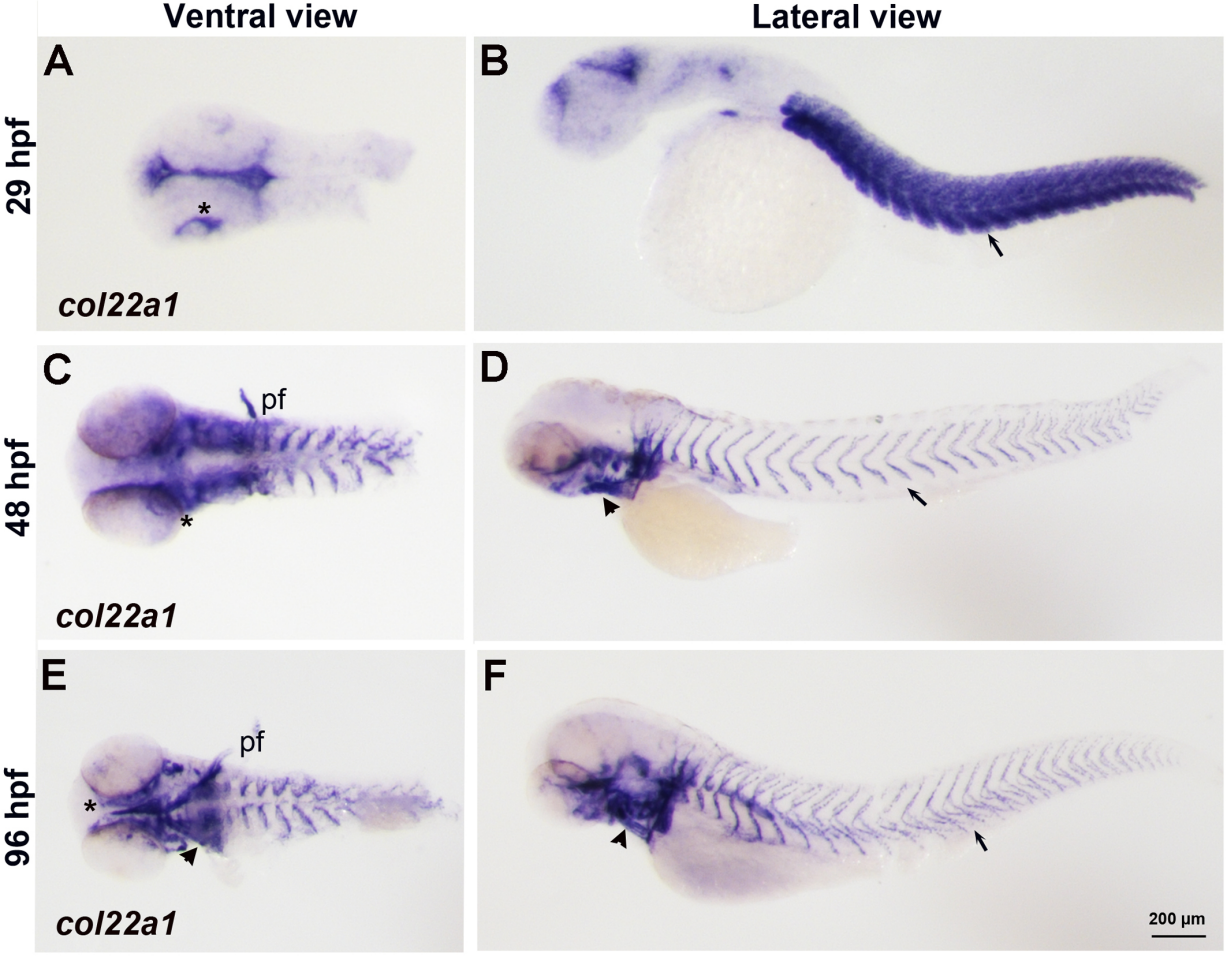
***col22a1* expression pattern analyzed by WISH.** (A-F) *col22a1* expression in flat-mounted embryos is viewed in ventral and lateral direction at 29 hpf (A,B), 48 hpf (C,D) and 96 hpf (E,F). The expression was detected in the somitic muscle (arrow, B) and at the myotendinous junction (arrows, D,F). *col22a1* expression was also detected around the eyes (asterisks), in the ears, pectoral fins (pf) and pharyngeal arches (arrowheads).

*col22a1* expression at the myoseptae and at the myotendinous junction of skeletal muscle in zebrafish embryos has been previously reported (Charvet et al., 2013). However, its expression outside the trunk region has not been characterized. To identify the expression pattern of *col22a1* in the cranial tissue, we performed whole-mount *in situ* hybridization (WISH) on zebrafish embryos at 29 h postfertilization (hpf), 72 hpf and 96 hpf (Fig. 1). The expression of *col22a1* was apparent around the eyes at 29 hpf and significantly increased between the medial region of the brain and the ventral side of the eyes starting at 48-96 hpf. The expression was also concentrated in pharyngeal arches and in pectoral fins. In addition, *col22a1* expression was strongly present in the skeletal muscle at 29 hpf and at the myotendinous junction at 48-96 hpf, as previously reported (Charvet et al., 2013).

**Fig. 2.**
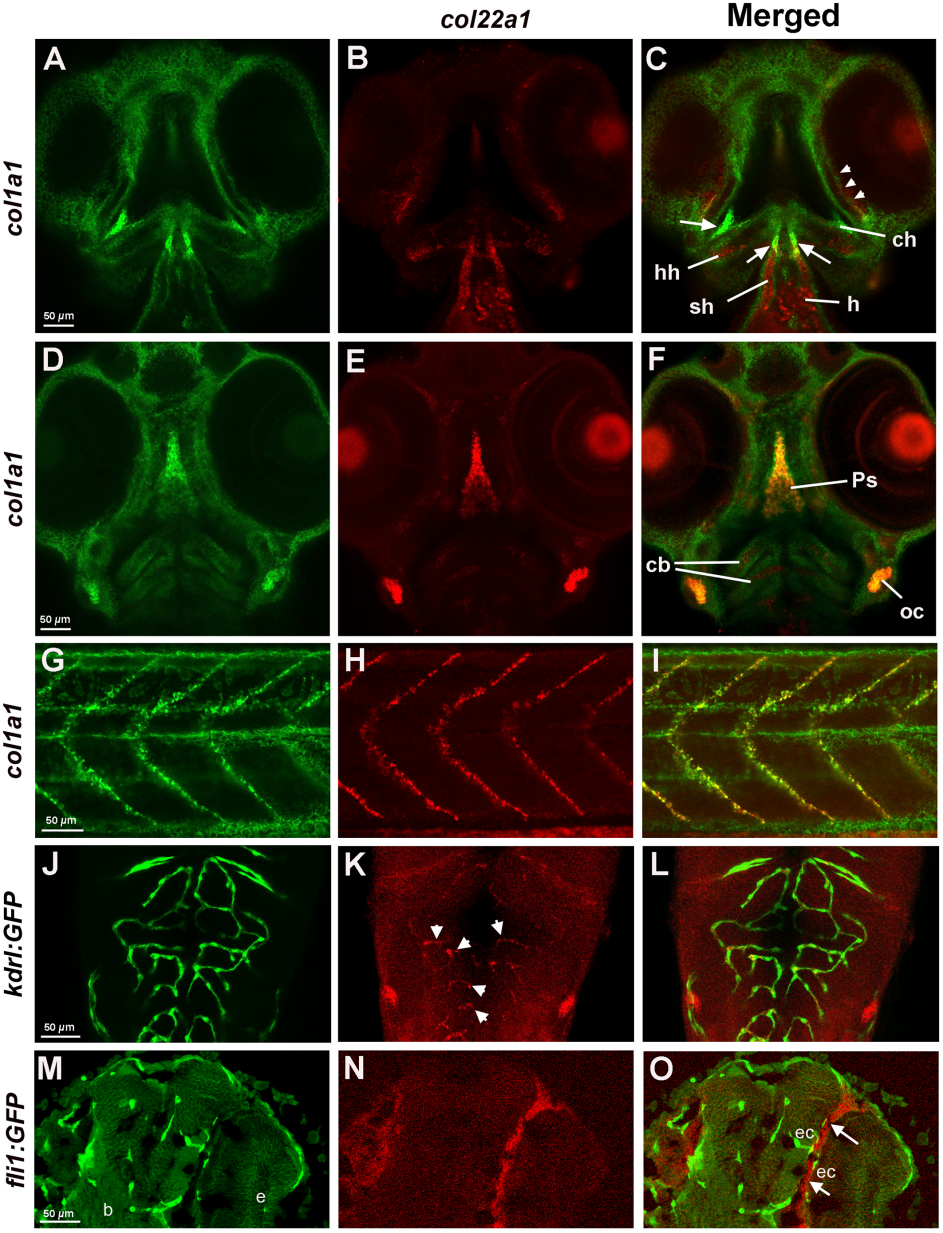
***col22a1* expression in the head and trunk region analyzed by FISH and FISH/immunofluorescence.** (A-I) Expression of *col22a1* (red) and *col1a1* (green) at 3 dpf, which commonly marks connective tissue. Expression was analyzed by HCR. In the transverse confocal sections of the cranial tissue (ventral view, A-F), *col22a1* expression is apparent in the periocular tissue (arrowheads, C), craniofacial muscle, including sternohyoideus (sh) and hyohyoideus (hh) muscles, parasphenoid bone (Ps), the heart (h), presumptive chondrocytes within ceratobranchial (cb) and ceratohyal arches (ch), and connective tissue within the developing ear (otic capsule, oc). Note that the expression of both markers overlaps within the parasphenoid bone, otic tissue, connective tissue around the eyes (arrowheads, C), and the tendon/ligament attachment sites of the sternohyoideus and hyohyoideus muscles (arrows, C). Within the myotendinous junction in the trunk region (G-I), all *col22a1*-expressing cells were also positive for *col1a1* expression. Note that *col1a1* also labels keratinocytes within the epidermis. (J-L) *col22a1* expression analyzed by HCR partially overlaps with vascular endothelial expression of *kdrl:GFP*. Cranial vasculature of whole-mounted embryos at 3 dpf was imaged dorsally by confocal microscopy. Anterior is to the top. Maximal-intensity projection of selected slices is shown. (M-O) Longitudinal cranial sections of embryos stained by WISH/immunofluorescence for *col22a1* (red) and vascular endothelial *fli1:GFP* (green) at 72 hpf. *col22a1* expression is apparent within the perivascular stromal cells (arrows, K). b, brain; e, eye; ec, endothelial cells. Note that these sections are located more ventrally compared with the vascular *col22a1* expression shown in J-L.

To characterize *col22a1*-expressing cells in the cranial tissues in greater detail, we performed two-color fluorescent *in situ* hybridization (FISH) using the hybridization chain reaction (HCR) (Choi et al., 2016) approach for *col22a1* and *col1a1* (also known as *col1a1a*), which is expressed in the connective tissue and fibroblast cells (Gistelinck et al., 2016). In the confocal sections, *col22a1* expression in embryos at 3 days postfertilization (dpf) was observed in the connective tissue around the eyes, craniofacial muscle, parasphenoid bone, the heart, cartilaginous cells within ceratobranchial, and ceratohyal arches and connective tissue within the developing ear (Fig. 2A-F). *col22a1* expression within the craniofacial muscle and heart did not overlap with *col1a1* expression, and *col22a1*-positive cells within the ceratobranchial arches were surrounded by *col1a1*-positive cells. However, expression of both markers overlapped within the parasphenoid bone, otic tissue, connective tissue around the eyes, and the tendon/ligament attachment sites of the sternohyoideus and hyohyoideus muscles. Within the myotendinous junction in the trunk region, all *col22a1*-expressing cells were also positive for *col1a1* expression (Fig. 2G-I). To analyze expression of *col22a1* relative to blood vessels, *Tg*(*kdrl:GFP*) embryos (Jin et al., 2005) were stained at 72 hpf for *col22a1* expression by HCR and imaged by confocal microscopy. *col22a1* expression in vascular endothelial cells of multiple cranial vessels was apparent (Fig. 2J-L). To image deeper cranial tissues, a conventional WISH followed by immunofluorescence of vascular endothelial-specific *fli1a:GFP* expression (Lawson and Weinstein, 2002) was performed. The longitudinal cryosections in the head region revealed the expression of *col22a1* within the fibroblast-like cells of the connective tissue adjacent to a subset of the cranial vasculature, particularly in the periocular region (Fig. 2M-O). Some flattened fusiform *col22a1*-expressing cells stained positive for Krt18 (also known as Krt18a.1), known to be expressed in optic nerve astrocytes and Muller glia (Koke et al., 2010) at 4 dpf (Fig. S1A-C). We also detected another subset of *col22a1*- and Krt18-positive cells that appeared cuboidal in shape. In addition, many *col22a1*-positive cells were located adjacent to the intermediate filament protein Vimentin at 3 dpf (Fig. S1D-F). Because a significant portion of the cranial connective tissue is derived from the neural crest (Bronner and LeDouarin, 2012), we tested colocalization of *col22a1* with *sox10:RFP*, which is known to be expressed in neural-crest-derived tissues (Simoes-Costa and Bronner, 2015). Indeed, *col22a1* expression overlapped with *sox10:RFP* expression in a subset of cells in cranial tissues adjacent to the eyes (Fig. S1G-I), and in the majority of cells in the pharyngeal arches (Fig. S1J-L), indicating that these cells are derived from the neural crest. In summary, *col22a1* expression at 3 dpf and 4 dpf was observed in a subset of vascular endothelial cells, pharyngeal muscle and perivascular fibroblast-like cells.

Several recent studies performed single-cell profiling of mouse brain and vascular adult or embryonic tissues. We analyzed *Col22a1* expression in the dataset obtained by single-cell profiling (scRNA-seq) of the adult brain vasculature (Vanlandewijck et al., 2018). Highest *Col22a1* expression was observed in perivascular fibroblast-like cells from adult mouse (Fig. S2A,B), while vascular endothelial cells showed much lower, but still significant, *Col22a1* expression. A different study performed scRNA-seq of postnatal mouse brain and spinal cord at postnatal day (P) 2 and P11 (Rosenberg et al., 2018). Average *Col22a1* expression among the combined brain and spinal cord clusters in the postnatal mice was highest in a group of ‘vascular and leptomeningeal cells 2’ (Fig. S2C), which are also positive for Slc6a13 and multiple other collagen homologs including Col1a1 and Col1a2, suggesting that these cells might be similar to the perivascular fibroblast-like cells reported by Vanlandewijck et al. (2018). Significant *Col22a1* expression was also noted in the ependyma and smooth muscle cells. Lower, but still significant, expression in the endothelial cells was also observed (Fig. S2C). These results argue that *Col22a1* in both zebrafish and mouse is expressed in perivascular fibroblast-like cells, as well as in vascular endothelial cells.

### *col22a1* deletion results in hemorrhages

**Fig. 3.**
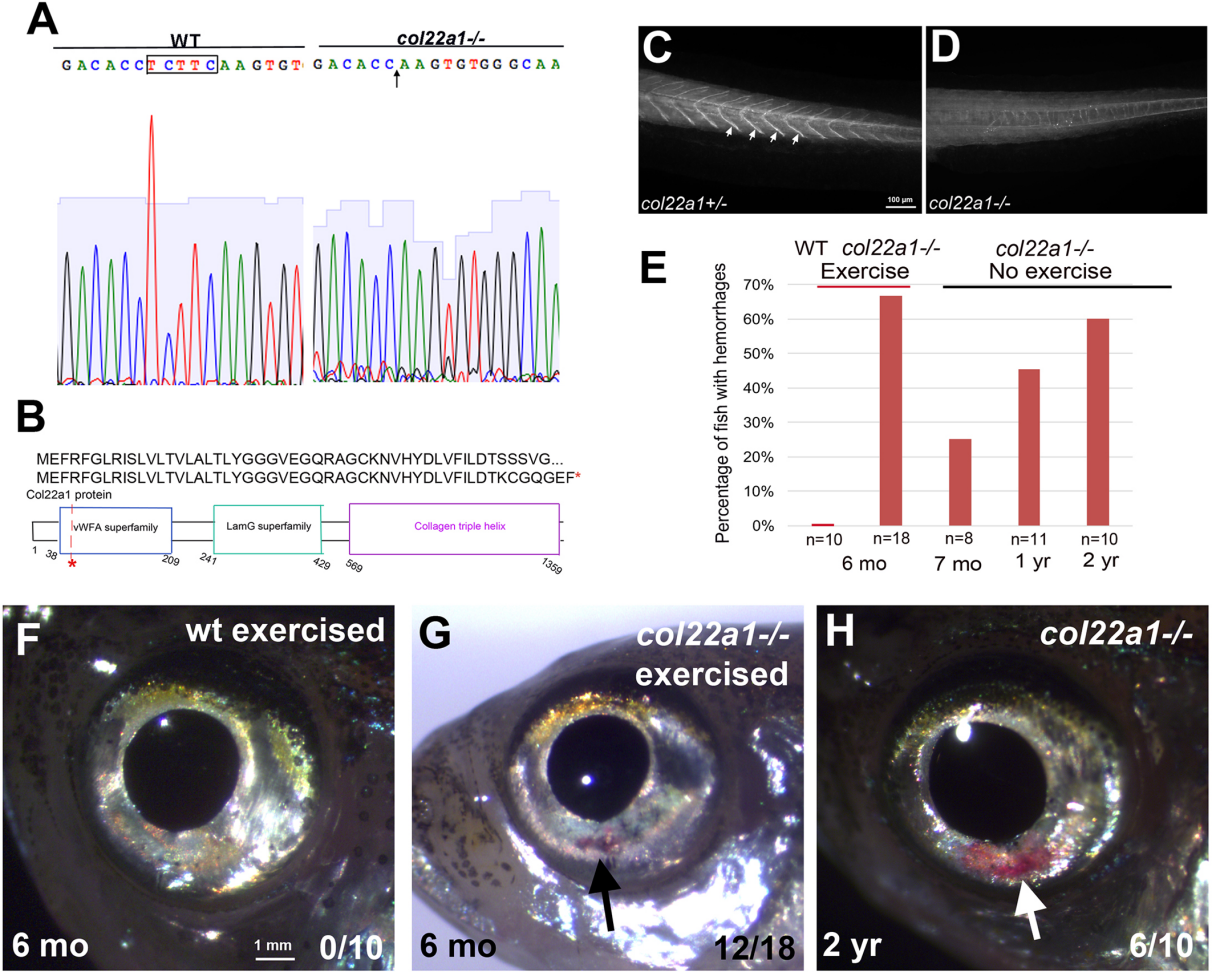
***col22a1^−/−^* mutant adults exhibit hemorrhages.** (A,B) Generation of *col22a1* zebrafish mutant line using TALEN. Sanger sequencing data of homozygous *col22a1* mutant show that there is a 5 bp deletion TCTTC (boxed; arrow in the right panel) that results in a frame shift (A) and a premature stop codon in the protein sequence (B). (C,D) Immunostaining against zebrafish Col22a1 at 3 dpf in *col22a1^+/−^* and *col22a1^−/−^* embryos. Staining is absent at the myoseptae in the *col22a1^−/−^* mutant (arrows) compared with the *col22a1^+/−^* embryo, suggesting that the mutation is likely null. Selected sections from *Z*-stack were merged using the Extended Focus feature within the Axiovision software (Zeiss). (E) The frequency of hemorrhages increases with age in *col22a1^−/−^* mutants, while exercise caused *col22a1^−/−^* mutants to exhibit the phenotypes at an earlier age. (F,G) A 6-month-old *col22a1^−/−^* adult experiences visible blood accumulation in the eyes after a 3-week course of intermittent forced exercise (black arrow, G), whereas the WT adult does not (F). (H) Without exercise, a 2-year-old *col22a1^−/−^* adult also exhibits visible blood accumulation in the eyes (white arrow).

To evaluate the function of *col22a1 in vivo*, a pair of transcription activator-like effector nuclease (TALEN) constructs targeting zebrafish *col22a1* was designed. We generated a mutant line that carries a deletion of five base pairs, which is predicted to result in a frameshift leading to the premature stop codon (Fig. 3A,B). Immunostaining for Col22a1 protein showed a complete loss of specific immunofluorescence at the myoseptae in the homozygous mutant embryos [Fig. 3C,D; quantified fluorescence intensity ratio in wild-type (WT) and mutant embryos was 20.3:1], suggesting that the mutant is likely null. No apparent phenotypes were observed in the homozygous or heterozygous *col22a1* mutant embryos and larvae, and they were viable to adulthood. A fraction of *col22a1* homozygous mutant adults developed hemorrhages around the eyes (Fig. 3H). The percentage of homozygous mutant adults that developed visible hemorrhages in the eyes increased as they aged, while heterozygous siblings did not show any apparent phenotypes. We observed that 25% of mutant adults displayed hemorrhages at 7 months, compared with 60% of mutants at 2 years of age (Fig. 3E). These data suggest that aging could be a factor potentially facilitating the progression of the disease in the adult fish. Thus, we hypothesized that application of forced excessive cardiac overload stress (Jean et al., 2012) would elicit a more frequent hemorrhagic phenotype in the mutants at a younger age. We completed a 3-week forced fast-speed intermittent swimming exercise protocol and analyzed the frequency of visible hemorrhages in the eyes, observing that 67% of 6-month-old homozygous mutants, who previously had no apparent phenotype, showed blood pools in their eyes following the exercise (Fig. 3E-G). No WT fish developed hemorrhages under these conditions.

**Fig. 4.**
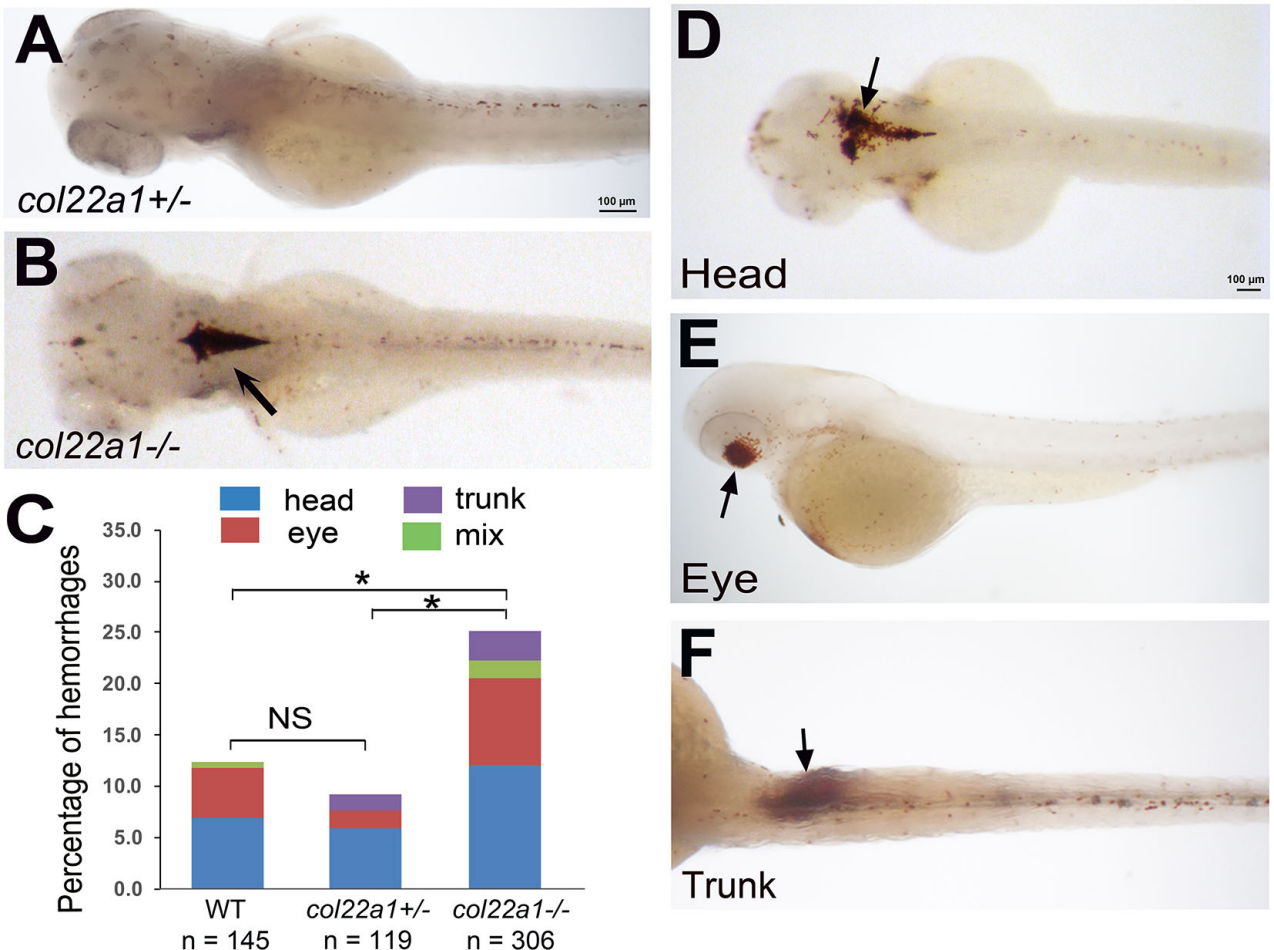
**Homozygous *col22a1^−/−^* embryos show increased percentage of hemorrhages when raised at increased temperature.** Embryos were stained with o-Dianisidine at 3 dpf. (A) The heterozygous *col22a1^+/−^* embryo shows small scattered blood cells in the head, while a large hemorrhage is apparent within the brain in the *col22a1^−/−^* embryo (arrow, B). (C) Compared with the WT and *col22a1^+/−^* embryos, a significantly higher percentage of *col22a1^−/−^* mutant embryos exhibit hemorrhages after heat stress. *P<0.005, determined by chi-square test. Embryo numbers were combined from three independent experiments (two for *col22a1^+/−^* embryos). The scoring numbers from each experiment are shown in Table S1. (D-F) Examples of hemorrhages (arrows) at different locations in heat-stressed *col22a1^−/−^* embryos.

Because cardiac stress induced hemorrhages in the adult fish, we speculated that similar environmental stress could also exacerbate the phenotype in the embryos. We employed a cardiovascular stress strategy as previously described (Barrionuevo and Burggren, 1999; Wang et al., 2014) by raising the homozygous and heterozygous *col22a1* mutants and WT embryos at an increased temperature of 33.5°C for two consecutive days starting at 24 hpf. The homozygous mutant embryos showed increased sensitivity to the heat stress and exhibited 2.5-fold higher frequency of intracranial hemorrhages compared with WT and heterozygous embryos (Fig. 4A-C). There were more heat-stressed *col22a1^−/−^* mutant embryos that exhibited blood accumulation in the head regions, eyes and trunks compared with heterozygous or WT embryos (Fig. 4D-F; Table S1). Interestingly, those hemorrhages occurred at or adjacent to the locations in which *col22a1* is expressed.

### *col22a1* deletion disrupts vessel integrity

**Fig. 5.**
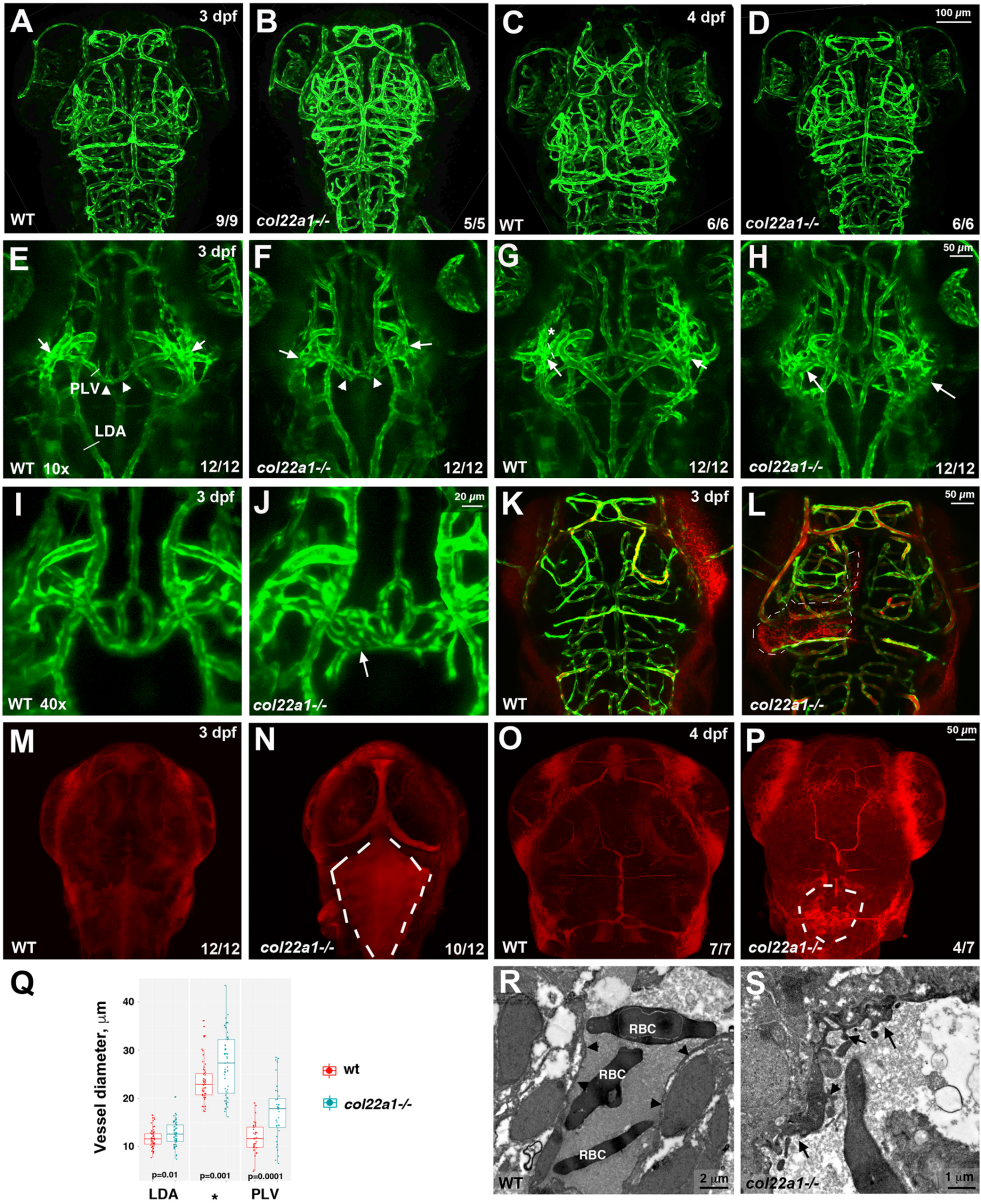
**Homozygous *col22a1^−/−^* mutant embryos show dilated and dysmorphic cranial vessels and have increased vascular permeability.** (A-D) Vascular patterning is not affected in *col22a1* mutants at 3 dpf and 4 dpf. Confocal maximal-intensity projections of live *kdrl:GFP* embryos, dorsal view, anterior is to the top. (E-J) Dilated and dysmorphic vessels are observed in *col22a1* mutants. Note the dilated and fused vessels at the vascular branch point in the periocular region (arrows) and the dilated and dysmorphic palatocerebral vein (PLV, arrowheads). Higher magnification (40×) images of the PLV are shown in I and J. Note that a portion of the PLV forms a plexus (arrow, J), which is not observed in WT embryos. Confocal maximal-intensity projections of selected slices of live *kdrl:GFP* embryos are shown, dorsal view, anterior is to the top. Cross-sections in the PLV, lateral dorsal aorta (LDA, E) and at the periocular branch point (asterisk, G) label the vessels selected for diameter measurement in Q. Different *Z*-stack projections of the same embryos are shown in E and G, and in F and H; I and J show different embryos. (K-P) Microangiography analysis of vascular permeability. FluoSpheres polystyrene microspheres (red, K,L) or low Mw (10 kDa) TRITC-dextran (red, M-P) were injected into the circulatory system of *kdrl:GFP*-positive WT and *col22a1^−/−^* embryos. Dorsal views of merged images (red, dextran/microspheres; green, *kdrl:GFP*) show rhodamine dye leakage at the brain ventricle and choroid plexus (dashed lines, N,P) and microsphere accumulation outside the vasculature (dashed lines, K,L) in *col22a1^−/−^* embryos at 3 dpf and 4 dpf. Maximal-intensity projections of confocal *Z*-stacks are shown. (Q) Vessel diameters of the LDA (E), PLV and the periocular branching point (asterisk, G) of heat-stressed *col22a1* mutants and wild-type (wt) embryos at 3 dpf. The measurements were made at three randomly selected points for each vessel in 12 mutant and wt embryos obtained in two independent experiments. LDA and periocular vessels were measured at both left and right sides where possible. The total number of measured points *n*=57 for LDA (s.d.=1.9 and 2.5 for wt and mutant embryos, respectively), *n*=54 and 57 for the periocular vessel (s.d.=4.3 and 6.5 for wt and mutant embryos, respectively), and *n*=36 for the PLV (s.d.=3.2 and 5.9 for wt and mutant embryos, respectively). *P*-values were calculated using Student's *t*-test between wt and *col22a1^−/−^* embryos for all measured points in all embryos. (R,S) TEM analysis of cranial vascular endothelium. Note the highly dysmorphic vascular endothelium (arrows, S) in *col22a1* mutants compared with normal vascular endothelium in WT embryos (arrowheads, R). RBC, red blood cells.

To determine whether vascular patterning was affected in *col22a1* mutants, they were crossed into a vascular-endothelial-specific Tg(*kdrl:GFP*) reporter line (Jin et al., 2005). No significant changes in the patterning of cranial vasculature were observed in heat-stressed *col22a1* mutant embryos at 3 dpf and 4 dpf (Fig. 5A-D). The diameter and gross appearance of the dorsally located cranial vasculature, including central arteries, did not show any significant differences between *col22a1* mutants and WT control embryos (Fig. 5A-D, and data not shown). However, significant differences in several deeper located vessels were apparent. In WT embryos, several vessels including the primordial hindbrain channel (PHBC), primary head sinus, primordial midbrain channel (PMBC) and palatocerebral vein (PLV) merge at periocularly located bilateral branch points (Isogai et al., 2001). Vasculature at these branch points was severely dilated in *col22a1* mutants, and individual vessels often appeared merged and could not be separated (Fig. 5E-H). The PLV, which returns blood into the bilaterally located PMBC and PHBC, had a highly uneven and dysmorphic appearance with multiple dilated areas, which often exhibited additional branches or plexus-like network (Fig. 5E,F,I,J). Average vessel diameters at this branch point and within the PLV were significantly increased in *col22a1* mutant embryos (Fig. 5Q). In addition, lateral dorsal aortae (LDA) showed increased diameter in *col22a1* mutants.

To analyze whether vascular permeability was affected in *col22a1* mutants, embryos were subjected to heat stress, starting at 1 dpf, and injected into the circulation system with polystyrene microspheres or 10 kDa tetramethylrhodamine isothiocyanate (TRITC)-dextran at 3 dpf or 4 dpf. Homozygous *col22a1* mutants showed increased microsphere and TRITC-dextran leakage from the cranial vessels (Fig. 5K-P), suggesting that dye extravasation was caused by an increase in vascular permeability.

We used transmission electron microscopy (TEM) to analyze the structure of the vascular endothelium in *col22a1* mutants and WT embryos. Based on TEM analysis, vascular endothelium in the intracranial vessels of *col22a1* mutants exhibited a highly uneven and dysmorphic appearance (Fig. 5R,S).

We further analyzed mural cell (smooth muscle and pericyte) coverage by confocal microscopy of *col22a1* mutants crossed into the *acta2:GFP* transgenic line. Expression of *acta2:GFP* in mural cells was not significantly changed in the majority of *col22a1^−/−^* embryos (Fig. S3A,B). In addition, expression of pericyte marker *pdgfrb* was not affected in *col22a1* mutant embryos (Fig. S3C-H). These results suggest that hemorrhages and vascular permeability defects are not caused by deficient mural cell coverage in *col22a1* mutants. Collectively, our data reveal that Col22a1 plays a critical role in maintaining vascular stability.

### Identification of mutant variants in *COL22A1* in the familial intracranial aneurysm (FIA) study

**Fig. 6.**
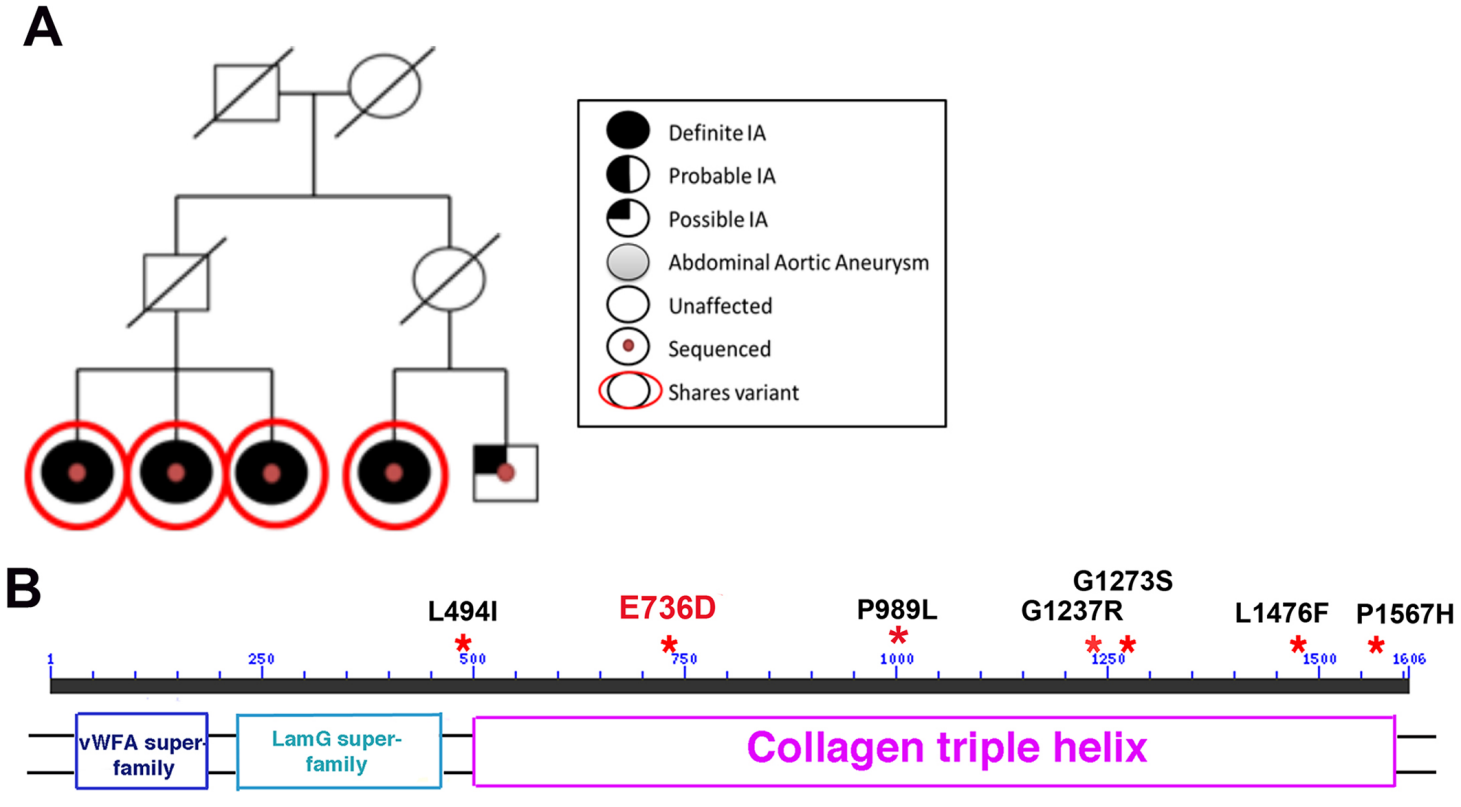
**Identification of COL22A1 SNP variants.** (A) The rs142175725 SNP predicted to result in the E736D amino acid substitution in COL22A1 segregates with affected individuals in the family. (B) Predicted protein structure of COL22A1 and specific amino acid position of SNPs (red asterisks) identified by WES and targeted genome sequencing. vWFA, von Willebrand factor; LamG, laminin G.

Next, we wanted to examine whether polymorphisms in *COL22A1* in human individuals might be associated with defects in vascular stability. In a recent FIA study, seven families and 45 individuals of European American descent were selected for WES to identify variants associated with IAs (Farlow et al., 2015). Although the study reported 68 variants that were retained after filtering and prioritizing by multiple selected criteria, multiple other additional mutant variants were identified in this study that were excluded from the final list. Among these variants, a SNP rs142175725 G/T, predicted to result in the E736D amino acid substitution, was identified within the collagen XXII (COL22A1) protein coding sequence among affected members in one of the families (family D) (Fig. 6A). Segregation of rs142175725 in all affected family members was calculated with a logarithm of odds (LOD) score of 1.11 (allele frequency, 0.0001264; minor allele frequency, 6.04×10^−5^). The random chance of seeing all members of family D carrying the SNP is 5.17×10^−8^. Although this LOD score is not high enough to definitely associate the SNP with IA phenotype, it is nevertheless suggestive of a potential association. The mutant variant is located in the triple-helix domain, which is commonly involved in oligomerization of monomeric collagen subunits. Glutamic acid E736 is 100% conserved between multiple vertebrates (Fig. 6B; Fig. S4). Targeted sequencing of 460 unrelated, familial FIA samples using multiplex PCR with primers specific to the exons within the *COL22A1* locus identified six additional variants that were present only in the affected individuals, but not found or found at a very low frequency in an Exome Aggregation Consortium (ExAC) non-Finnish European population (Fig. 6B; Table S2). All these SNPs are 100% conserved between multiple mammalian organisms, and three of them are conserved between humans and zebrafish (Fig. S4, and data not shown). In addition, all the SNPs are predicted to be ‘probably damaging’, according to PolyPhen-2 analysis (Adzhubei et al., 2010), and four of them are predicted to be damaging by SIFT (Ng and Henikoff, 2003) (Table S2). Two of the identified variants have mutations affecting glycine within the Gly-X-Y triple helix repeats (Fig. S4). Affected individuals were heterozygous for all of these variants, suggesting that these SNPs could have a dominant effect. These results suggested a possibility that mutations in *COL22A1* could be associated with increased risk of IAs. We then tested experimentally whether the E736D mutant variant affected COL22A1 function in a zebrafish model.

### Inducible overexpression of the human E736D COL22A1 variant increases the incidence of hemorrhages

**Fig. 7.**
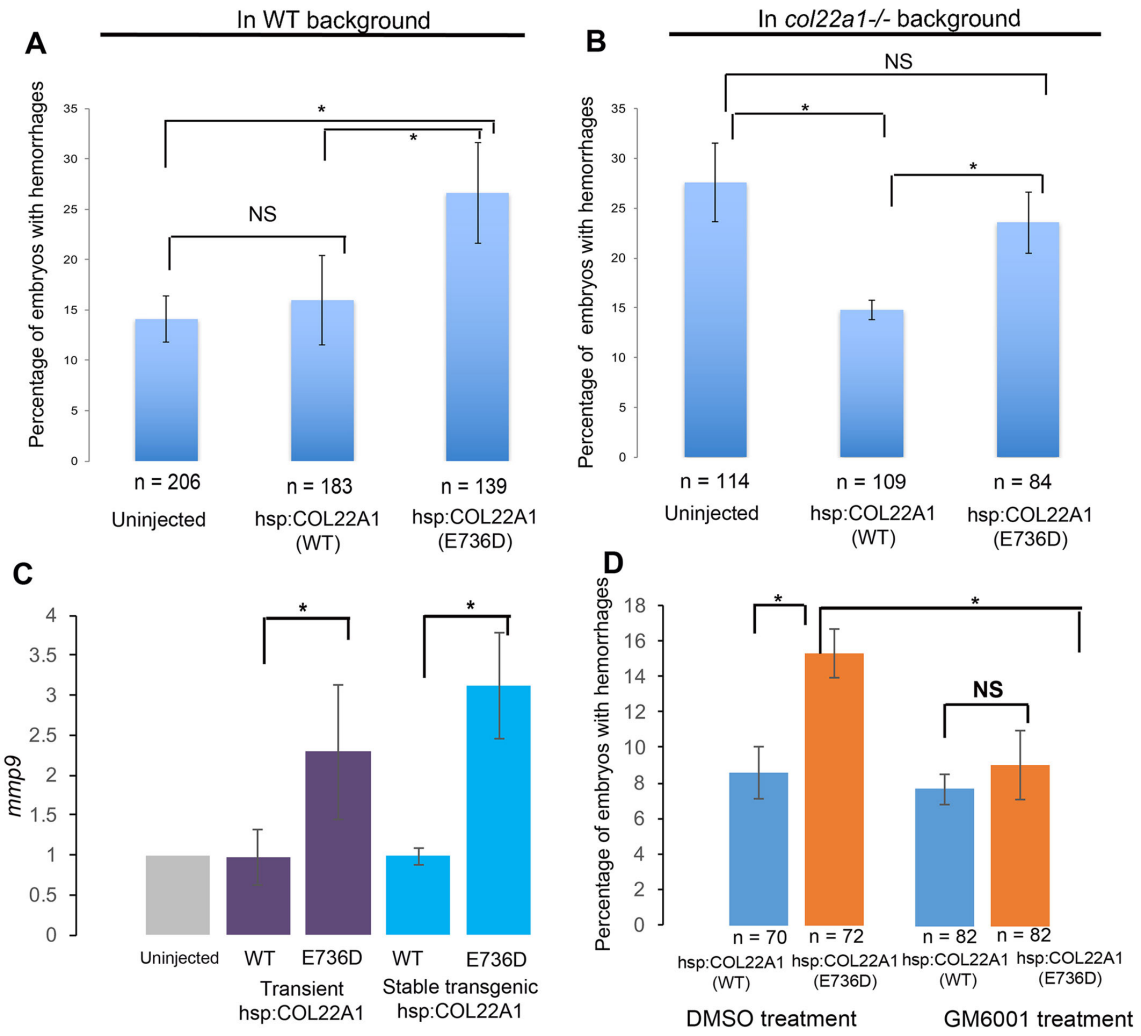
**The E736D mutation affects COL22A1 function and results in an increased percentage of embryos with hemorrhages.** (A) Transient overexpression of the human E736D variant of COL22A1 causes hemorrhagic phenotypes in WT embryo background. Compared with the uninjected embryos and WT *hsp70:COL22A1* plasmid DNA-injected embryos, the human E736D variant *hsp70:COL22A1* DNA-injected embryos exhibited a higher percentage of hemorrhages after *COL22A1* expression was induced by heat shock. (B) The human E736D *COL22A1* construct fails to rescue the zebrafish *col22a1^−/−^* mutant phenotype. In contrast, heat-shock-inducible overexpression of human WT *COL22A1* partially rescues the hemorrhagic phenotype in *col22a1^−/−^* mutant embryos. (C,D) MMP9 mediates hemorrhage formation in E736D *COL22A1*-overexpressing embryos. (C) qPCR analysis of *mmp9* expression in control uninjected embryos, in embryos injected with human WT and E736D *hsp70:COL22A1* constructs, and in stable transgenic WT and E736D *hsp70:COL22A1* embryos following the heat shock. There was an increase in *mmp9* expression in E736D *COL22A1*-overexpressing transgenic embryos. The level of expression in uninjected embryos was normalized to 1. (D) Inhibition of MMPs with a chemical GM6001 treatment rescues the hemorrhagic phenotype in human E736D *hsp70:COL22A1*-overexpressing embryos. There was a reduced percentage of embryos with hemorrhages among GM6001-treated embryos that were injected with E736D *hsp70:COL22A1* DNA construct compared with DMSO-treated embryos that were injected with the same construct. *P<0.05; NS, not significant; determined using Student's *t*-test. *n*, number of embryos used in the experiments. Error bars represent s.e.

The four affected family members from the WES study are heterozygous for the E736D variant, suggesting a dominant model of genetic inheritance. Thus, we conducted an overexpression experiment in zebrafish embryos to test whether E736D substitution exhibits a dominant deleterious effect. We injected a construct that carries either mutant or WT human *COL22A1* fused to the self-cleaving viral peptide 2A and mCherry under a heat-shock-inducible promoter into embryos at one-cell stage (Fig. S5A). At 23 hpf, embryos were heat shocked and then incubated at 33.5°C to induce heat stress for 2 days. Injected embryos were screened for mCherry fluorescence, an indicative signal for human COL22A1 overexpression (Fig. S5B,C), and analyzed for hemorrhages by heme staining with o-Dianisidine. Inducible overexpression of the human E736D COL22A1 variant in the WT background resulted in significantly more frequent hemorrhages, similar to zebrafish *col22a1^−/−^* mutant embryos (Fig. 7A). In contrast, the induction of human WT COL22A1 expression did not provoke a significant increase in the percentage of embryos with hemorrhages when compared with uninjected embryos.

We next assessed the functional activity of both human WT COL22A1 and E736D mutant variants by testing their ability to rescue the hemorrhagic phenotype present in the heat-stressed *col22a1^−/−^* embryos. As expected, overexpression of the WT human COL22A1 partially rescued the phenotype of mutant embryos (Fig. 7B). Conversely, overexpression of the inducible human E736D variant failed to rescue the phenotype in the injected *col22a1^−/−^* embryos. This combination of experiments argues that the E736D variant results in non-functional COL22A1 protein and has a dominant effect, which interferes with the function of the WT allele.

### The E736D COL22A1 variant causes hemorrhagic incidents by upregulating *mmp9* expression

We next investigated how COL22A1 deficiency mediates hemorrhagic phenotypes. Previous studies showed increased expression of metalloproteinases (MMPs) in the patient tissues with IAs (Kim et al., 1997). We performed quantitative real-time PCR (qPCR) to examine changes in *mmp9* and *mmp13* expression in the heat-shocked human E736D mutant and WT COL22A1-overexpressing embryos. At 3 dpf, when the hemorrhages are typically apparent, we observed an upregulation of *mmp9* expression in both transiently overexpressing E736D COL22A1 embryos and transgenic embryos with a stable *hsp70:COL22A1* integration, whereas *mmp13* expression remained unchanged (Fig. 7C; Fig. S5D,E, and data not shown), suggesting a potential contribution of *mmp9* to the pathology of IAs in zebrafish. In order to test whether the inhibition of MMPs could alleviate the hemorrhagic phenotype in the E736D COL22A1-overexpressing embryos, we treated the injected E736D embryos with a chemical MMP inhibitor GM6100 (Shichi et al., 2011). MMP inhibition by GM6100 led to a significant reduction in the frequency of hemorrhages among human E736D COL22A1-overexpressing embryos (Fig. 7D). Together, our findings provide evidence that COL22A1-dependent hemorrhagic phenotypes could be mediated by upregulation of *mmp9* expression, and suggest that MMP inhibitors could be effective for clinical therapy in patients with *COL22A1* mutations.

### The E736D COL22A1 variant stimulates endoplasmic reticulum (ER) stress response

**Fig. 8.**
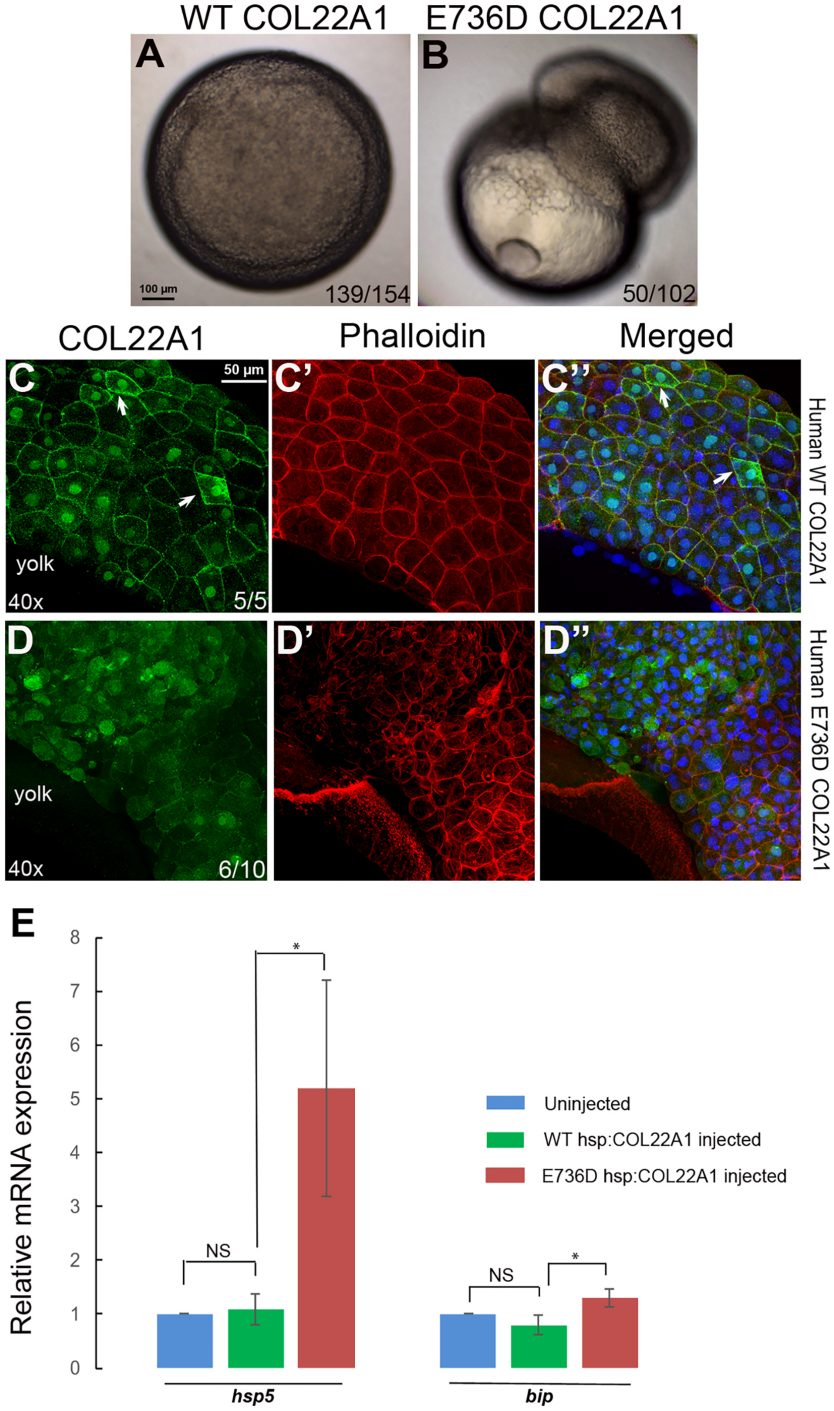
**Overexpression of human E736D *COL22A1* mRNA causes cell detachment during gastrulation, while the protein is retained in the cytoplasm leading to an increase in ER stress.** (A,B) Cells fail to undergo epiboly and detach from the yolk in the E736D *COL22A1* mRNA-injected embryo (B) during gastrulation compared with the human WT *COL22A1* mRNA-injected embryo (A). (C-D″) Human E736D COL22A1 protein is retained in the cytoplasm during gastrulation, whereas WT COL22A1 is secreted into the extracellular space (arrows). Confocal microscopy analysis of human COL22A1 protein immunofluorescence (green, C,D) and phalloidin staining (red, C′,D′). Blue, nuclear DAPI staining. (E) Aggregation of human mutant COL22A1 in the cytoplasm induces ER stress. qPCR confirms the elevation of ER stress markers *hsp5* and *bip* in human E736D *COL22A1* mRNA-injected embryos compared with the human WT mRNA-injected embryos. Expression in the uninjected samples was normalized to 1. *P<0.05, determined using Student's *t*-test. Error bars represent s.d.

We explored whether the human E736D variant is detrimental to the embryos at the cellular level. We injected mRNA of either human WT or mutant *COL22A1* into zebrafish embryos at one-cell stage. Injection of mutant E736D mRNA caused dramatic defects during embryonic gastrulation, interfering with the epiboly movement in 49% of embryos. Cells in the blastomere often detached from the yolk and lysed (Fig. 8A,B). Such a phenotype was observed only in E736D *COL22A1* mRNA-injected embryos; overexpression of the same dose of WT human *COL22A1* mRNA did not cause any apparent defects in the majority of embryos. To determine the cause of this phenotype, we analyzed collagen secretion by performing immunofluorescent staining for human COL22A1 in the injected embryos. The confocal images capturing cells at gastrulation stage show that the mutant variant appears largely intracellular compared with the WT form that was secreted (Fig. 8C-D″). This suggests that the mutant variant fails to be secreted into the extracellular environment. The failure of secretion could be caused by protein misfolding, which would lead to an ER stress response due to the accumulation of misfolded proteins. Indeed, qPCR demonstrated elevated expression of *hsp5* and *bip* markers, associated with ER stress (Ron and Walter, 2007; Walter and Ron, 2011) (Fig. 8E). We further analyzed the effect of the E736D variant on the expression of ECM components *in vitro* by transiently transfecting human fibroblast cells with the DNA constructs that either carry human WT or E736D mutant COL22A1 tagged with His/Myc. Compared with cells that were transiently transfected with human WT COL22A1, cells with the E736D COL22A1 mutant construct showed more intense intracellular COL22A1 staining, in addition to the reduction of laminin staining, suggesting that the defective COL22A1 protein is retained in the cytoplasm (Fig. S6). The loss of laminin could be caused by the aggregation of the mutant protein inside the cells, which leads to ER stress and interferes with the synthesis and secretion of other ECM molecules. Collectively, these results suggest that the E736D COL22A1 mutant variant might interfere with protein folding, which would result in the ER stress response and misexpression of the ECM components, ultimately leading to cellular death.

## DISCUSSION

In this study, we have demonstrated a functional role for Col22a1 in maintaining vascular stability in the zebrafish model system. We also identified mutant variants in *COL22A1* that are associated with IAs in affected individuals. Overexpression analysis shows that the E736D variant has a deleterious effect on Col22a1 function and likely interferes with the protein folding. Taken together, our results suggest that mutations in *COL22A1* could be one of the causes of IAs in humans. However, further genetic analysis using larger patient samples is needed to confirm the association of mutations in *COL22A1* with intracranial aneurysms.

Our results show that *col22a1* is expressed in vascular endothelial cells of the cranial vasculature, as well as in connective and craniofacial muscle tissue. Expression of *col22a1* was observed in perivascular fibroblast-like cells, and at a lower level in vascular endothelial cells, in both zebrafish and mouse. Significant Col22a1 expression in mouse embryos was also noted in the ependyma based on the single-cell data analysis (Rosenberg et al., 2018). Interestingly, zebrafish *col22a1* expression partially overlapped with Krt18 expression, which is known to be expressed in rat ependymal cells (Kasper, 1992). These data suggest that many of the *col22a1* expression domains are similar between zebrafish and mammals, and it is likely that *col22a1* function is also evolutionarily conserved.

We demonstrated that *col22a1* homozygous mutant adults, but not their heterozygous siblings, exhibit increased frequency of hemorrhages. The percentage of *col22a1^−/−^* adult fish that experienced the hemorrhagic phenotype increased with age or upon cardiovascular stress. In addition, *col22a1* mutant embryos exhibit increased percentage of hemorrhages, likely caused by cardiovascular stress due to increased incubation temperature. Although we did not observe berry-like aneurysms in *col22a1* mutants, they exhibited vessel dilations, which are commonly observed in fusiform aneurysms. Some of the vessels possess irregular shapes. These vessels have enlarged lumen, along with irregular and variable width of the vascular wall. Intriguingly, TEM analysis detected dysmorphic vascular endothelium in *col22a1* mutant embryos. Although the pericytes and smooth muscle cells did not appear significantly affected, it is possible that vascular permeability defects in *col22a1* mutant phenotypes are caused by an altered structure or composition of the ECM. The exact cause of *col22a1* mutant phenotype will require further investigation.

A previous study has shown that morpholino-mediated gene knockdown of *col22a1* function in zebrafish embryos results in defective myotendinous junctions (Charvet et al., 2013). Here, we did not observe any apparent defects in skeletal muscle or myotendinous junctions in genetic *col22a1* mutant embryos or adults. As evident from the loss of immunostaining, the *col22a1* mutation results in the complete, or nearly complete, loss of Col22a1 protein, which suggests that the previously reported phenotype was possibly caused by morpholino off-target effects (Eisen and Smith, 2008; Kok et al., 2015). However, there is recent evidence for genetic compensation occurring in genetic mutants, but not in morphants, that may also explain the absence of defective muscles in *col22a1* mutants (Rossi et al., 2015). Instead, we witnessed the loss of cranial vessel integrity in the homozygous mutant embryos and adults following cardiovascular stress.

We also demonstrated that expression of the human COL22A1 familial E736D variant interferes with COL22A1 function and can lead to cell death. Furthermore, inducible overexpression of human E736D COL22A1 variant recapitulates hemorrhagic phenotypes that are seen in the *col22a1* mutant zebrafish at 3 dpf. In early development and *in vitro*, the E736D COL22A1 human variant fails to be secreted and is likely retained in the cytoplasm. Increased expression of ER stress markers suggests that the E736D variant affects COL22A1 protein folding. Misfolded protein likely accumulates in the ER, triggering the misfolded protein response pathway that results in the elevation of ER stress. Ultimately, this may lead to misexpression of other ECM proteins, such as laminin, and finally results in cell death. This explains how early E736D overexpression results in a lethal phenotype during gastrulation stages. Future studies will be focused on defining how COL22A1 might mediate the formation of aneurysms.

Several studies have described the pathophysiological involvement of MMP9 in intracranial aneurysms in human patients and the relationship between MMP9 and collagen fibrils (van Doren, 2015; Hashimoto et al., 2006). In zebrafish embryos, we demonstrated that overexpression of the human E736D COL22A1 variant resulted in *mmp9* upregulation. This suggests that *mmp9* functions downstream of COL22A1 to mediate hemorrhagic phenotypes. Indeed, upon inhibition of MMPs, the frequency of hemorrhagic phenotypes caused by inducible overexpression of human mutant COL22A1 is reduced. Our finding that *mmp9* could function downstream of *col22a1* is consistent with the reported role of MMP9 in the formation of intracranial aneurysm in mice (Nuki et al., 2009). The mice with induced hypertension developed IA with either thick or thin vascular wall, in addition to disorganized elastic lamina (Nuki et al., 2009). At present, we do not know whether hypertension was present in the COL22A1-overexpressing zebrafish embryos, but the documented phenotypes resemble typical features of IA. Continued studies are required to determine whether COL22A1 and MMP9 interact directly or indirectly to influence the pathology of the disease.

In summary, our results argue that COL22A1 functions to maintain vascular stability. They further suggest that mutations in *COL22A1* could be one of the causes of intracranial aneurysms in humans. Further studies are warranted to examine the frequency of *COL22A1* variants in familial and sporadic IA. Future studies could utilize the zebrafish as a model system for screening chemical compounds that could reduce the frequency of hemorrhages and promote the development of new treatments for IAs.

## MATERIALS AND METHODS

### Zebrafish lines and zebrafish embryo handling

The following zebrafish lines were used in experiments: *Tg(fli1a:EGFP)^y1^* (Lawson and Weinstein, 2002), *Tg(kdrl:GFP)^s843^* (Jin et al., 2005), *Tg(acta2:EGFP)^ca7^* (Whitesell et al., 2014), *Tg(sox10(7.2):mRFP)* (Kucenas et al., 2008), *TgBAC(pdgfrb:EGFP)^ncv22^* (Ando et al., 2016) and AB WT (Zebrafish International Resource Center). Embryos were incubated at 28.5°C or 33.5°C as described in the text and staged using criteria previously described (Kimmel et al., 1995). Embryos beyond 24 hpf were treated with 0.003% 1-phenyl-2-thiourea (PTU) to inhibit the formation of pigment. Zebrafish adults were housed in a re-circulating system (Pentair). The fish were maintained under the Institutional Animal Care and Use Committee-approved protocol.

### Generation of the *col22a1* TALEN mutant line

We used TALENs (Bedell et al., 2012; Zu et al., 2013) to generate a *col22a1^ci16^* mutant that carries a five-nucleotide deletion at exon 2 (ENSDARG00000078899, Zv9). TALEN constructs were assembled using a Golden Gate TALEN kit (Cermak et al., 2011) and correspond to the following sequences: TAL-L, NH NI NG HD NG NH NH NG NI NG NG HD NI NG NG NG NG NH; TAL-R, HD NI NI NI NI NG NG HD NG HD HD NG NG NH HD HD HD NI. These dimers recognize the targeted *col22a1* sequence (italicized) within the spacer fragment (capitalized) TGATCTGGTATTCATTTTG*gacacctcttcaagtg*TGGGCAAGGAGAATTTTGA. Founders were screened for 5 bp deletion by genotyping using the PCR primers (Supplementary Information) and *Ear*I restriction enzyme to distinguish the mutant from the WT bands.

### Cell line usage and transfection

Human primary lung fibroblast cells CCD-19 Lu [American Type Culture Collection CCL-210; a gift from Dr Timothy LeCras, Cincinnati Children's Hospital Medical Center (CCHMC), Cincinnati, OH, USA] were kept in a humidified incubator at 37°C and 5% CO_2_. Cells were incubated overnight until they reached 80-90% confluency. Cell were then treated with 0.25% trypsin/EDTA and seeded in a 12-well plate with microslips in 1% gelatin. When they reached 50-70% confluency, transient transfection was performed by lipofectamine following the protocol as described (Thermo Fisher Scientific, 116688027).

### WISH and HCR

A complementary DNA (cDNA) clone corresponding to the partial zebrafish *col22a1* sequence in pExpress-1 was obtained from Open Biosystems (Thermo Fisher Scientific). The digoxigenin-labeled *col22a1* riboprobe was generated using SP6 RNA polymerase (Promega, P407A) from *Eco*RV-linearized *col22a1*-pExpress-1. *pdgfrb* probe was generated as previously described (the construct was generously donated by Appel Lab) (Wang et al., 2014). WISH was performed as previously described (Jowett, 1999). Embryos were washed in 4% paraformaldehyde (PFA; Electron Microscopy Sciences, 15714) followed by washes in PBS+0.1% Tween 20 (PBST). The embryos were incubated in RIMS clearing reagent for 1 h (30 g Histodenz in 40 ml 0.2 M phosphate buffer with 50 µl Tween 20 and 10 mg sodium azide) and then washed extensively with PBST. Processed embryos were dehydrated in 100% methanol for storage at −20°C to improve contrast, then rehydrated in 1× PBS and mounted in 3% methylcellulose under a coverslip for imaging. Images were taken using a Zeiss M2BioV12 stereomicroscope.

HCR (version 3) for *col22a1* and *col1a1* co-expression was performed as described (Choi et al., 2018). HCR probes were synthesized by Molecular Technologies at Beckman Institute, California Institute of Technology, Pasadena, CA, USA. For co-expression of *kdrl:GFP* and *col22a1* analysis, GFP-positive embryos were fixed at 3 dpf. Subsequently, they were processed using *col22a1* HCR probe. GFP fluorescence was still apparent after HCR. Embryos were imaged using Nikon A1 confocal microscope at the CCHMC Confocal Imaging Core.

### Immunochemistry

The following primary antibodies were used: zebrafish anti-Col22a1 (1:200; kindly donated by F. Ruggiero) (Charvet et al., 2013), goat anti-human COL22A1 (1:50 for embryo tissue, 1:200 for fibroblast cells N17; Santa Cruz Biotechnology, sc87647), rabbit anti-Laminin (1:100; Sigma-Aldrich, L9393), mouse anti-α-Tubulin bovine (1:400; Life Technologies, A11126), mouse anti-Vimentin (1:50; Thermo Fisher Scientific, MA5-11883), rabbit anti-DsRed that can recognize RFP (1:100; Takara, Living Colors, 632496), Alexa Fluor 488 Phalloidin (Thermo Fisher Scientific, A12379), mouse anti-KRT18 (1:27; Abgent, AT2655a), rabbit anti-GFP (1:200; Life Technologies, A21311), Alexa Fluor anti-rabbit 594 (1:200; Life Technologies, R37117), Alexa Fluor anti-goat 488 (1:200; Life Technologies, A11055) and Alexa Fluor anti-mouse 488 (1:200; Life Technologies, A11001). Prolong Diamond Antifade Mountant mounting medium was used to analyze Tubulin staining; VectaShield mounting medium with 4′,6-diamidino-2-phenylindole (DAPI) was used to analyze Laminin staining. Images of Col22a1 immunofluorescent staining in WT embryos and *col22a1* mutants were quantified using Fiji (ImageJ) software (National Institutes of Health). Averages of fluorescence intensity in five randomly selected points at the myoseptae region were calculated with Fiji followed by the background subtraction. The relative fluorescence intensity ratio in WT embryos and *col22a1* mutants was 20.3:1.

### WISH/immunofluorescence on sections

WISH-processed embryos were fixed in 4% PFA for 30 min and washed in PBST before proceeding through the embedding process as previously described (Ton and Iovine, 2013). The embryos were cryosectioned using a Microm HM 550 cryostat. The slides were air dried for 2 h or overnight. Slides were then dehydrated in 100% ethanol for 5 min then air dried for 5 min. The sections were rehydrated with PBS for 5 min twice. Slides were then washed with PBST for 5 min followed by a wash with the blocking solution (1% bovine serum albumin in PBST) for 1 h. The slides were incubated in the blocking solution with primary antibody overnight at 4°C. The blocking solution with secondary antibodies was then applied to the slides for 2 h at room temperature. Images were captured using a Nikon confocal A1Rsi inverted microscope (40× objective) at the CCHMC Confocal Imaging Core facility. Confocal imaging of NBT/BCIP substrate fluorescence was performed as previously described (Schumacher et al., 2014).

### TEM

For TEM analysis, zebrafish larvae were fixed in 2.5% fresh glutaraldehyde in 100 mM cacodylate buffer overnight at 4°C. The larvae were then washed in cacodylate buffer and postfixed in 1% tannic acid. They were next transferred to 1% osmium tetroxide and then embedded in Embed-812 resin (Electron Microscopy Sciences) following dehydration in an acetone series. Ultrathin sections (100 nm) were cut, set on single-slot or 200-mesh copper grids and imaged on a JEOL JEM 1010 (JEOL USA, Peabody, MA, USA).

### Cardiovascular stress on zebrafish adults and embryos

Adult mixed sex WT and *col22a1* TALEN homozygous mutants were examined for the absence of any external hemorrhages before undergoing excessive forced swimming exercise in the 5 l swimming tunnel (Loligo Systems, SW10050). The swimming speed was adjusted manually at 750 V. Five fish were exercised for two 60-min sessions per day, 5 days per week for 3 weeks.

PTU-treated embryos were kept at 28.5°C. When the embryos reached 24 hpf, they were transferred to a 33.5°C incubator and kept for two consecutive days. At 72 hpf, embryos were processed for heme staining using o-Dianisidine as previously described (Detrich et al., 1995).

### Histology

Paraffin blocks were cut into 10 μm sections on a Leica RM 2125 microtome. Sections were then floated on a water bath before being placed onto slides. The slides with sections were oven baked at 65°C to dry. Sections were then loaded onto a Ventana Symphony auto-stainer (Ventana Medical Systems, Tucson, AZ, USA) for Hematoxylin and Eosin (H&E) staining using the standard protocol in the default settings. This system combines processes of baking, de-waxing, staining and cover slipping, producing permanently mounted and H&E-stained sections for histologic/microscopic assessment.

### Microangiography

Heat-stressed live 72 hpf or 96 hpf embryos were injected into the perivitelline space with 10 kDa TRITC-dextran (Molecular Probes, Life Technologies, D1817), or with FluoSpheres carboxylate-modified microspheres 0.02 µm, red fluorescent (Molecular Probes, Invitrogen, F8786), and were embedded in low-melting-point agarose and immediately imaged using a Nikon A1R LUN-V inverted microscope with 20×/0.75 Multi immersion objective at the CCHMC Confocal Imaging Core facility. The maximal-intensity projection was calculated using Fiji software. The diameter of blood vessels was measured using Fiji software at three selected points in each representative vessel in 12 mutant and 12 wild-type embryos obtained in two independent experiments.

### Human *COL22A1* mRNA overexpression

The Gateway entry construct pENTR223.1 containing the full-length human *COL22A1* sequence was obtained from Open Biosystems (Thermo Fisher Scientific). The construct was subcloned using the Gateway cloning method (Thermo Fisher Scientific) into pCS2 Dest vector (Villefranc et al., 2007). A site-directed mutagenesis kit (Agilent Technologies) was used to perform G-to-T substitution corresponding to the E736D mutation within the human *COL22A1* coding sequence present in pCS2-Dest vector. The *COL22A1*-pCS2-Dest vector was linearized with *Kpn*I and was *in vitro* transcribed using an SP6 mMessage mMachine kit (Ambion, AM1340). At one-cell stage, 380 pg of either WT or mutant E736D mRNA was injected into zebrafish embryos. Embryos were scored at the germ ring to shield stage under bright-field imaging or fixed in 4% PFA overnight for immunofluorescence.

### Generation of the inducible overexpression human *COL22A1* WT and E736D mutant constructs

To make *tol2*-*hsp70:COL22A1-2A-mCherry*; *α-crystallin:DsRED-tol2*, an LR reaction that uses site-specific Gateway recombination technology was performed with the following constructs: *tol2*-*hsp70:COL22A1-2A-mCherry*, an entry vector with *hsp70* promoter (p5E-*hsp70*) (Kwan et al., 2007); a full-length human *COL22A1* gene in pDONR223 (Invitrogen) that has attL1 and attR1 sites; a 3′ entry vector with 2A-mCherrypA (Covassin et al., 2009); and a destination vector pDestTol2p2A with *α-crystallin:DsRED* (Mandal et al., 2013). To create *col22a1-His-myc*, an LR reaction was performed using human *COL22A1* in pDONR223, and pEZHis-myc (generously provided by Dr Aaron Zorn, Cincinnati Children's Hospital). Mutant E736D *COL22A1* constructs were generated by a Quick Change II XL Site-Directed Mutagenesis kit using *tol2*-*hsp70:COL22A1-2A-mCherry* and *COL22A1-His-myc* constructs as templates.

### *COL22A1* conditional overexpression

WT *Tg(hsp70:COL22A1-mCherry)^ci18^* and *Tg(hsp70:E736D COL22A1-mCherry)^ci31^* zebrafish lines were generated by injecting 25 pg *tol2*-*hsp70:COL22A1-mCherry*; *α-crystallin:DsRED-tol2* with 25 pg *tol2* mRNA into one-cell-stage zebrafish embryos. Transgenic embryos were identified by heat-shock-inducible mCherry or *α-crystallin:DsRED* expression, and groups of transgenic and control embryos were raised at 28.5°C, heat shocked twice at 24 hpf and 48 hpf for 1 h at 37°C, and returned to 28.5°C to be raised and analyzed for the presence of hemorrhages. Founders were screened for successful transmission of mCherry and *DsRED.*

### MMP inhibitor chemical treatment

GM6001 (Millipore, CC1010) was purchased as a 2.5 mM stock in dimethyl sulfoxide (DMSO). Transiently overexpressing Tg(*hsp70:col22a1-mCherry*) and stable transgenic embryos were treated with 25 μM GM6001 in embryo water starting at 24 hpf as described previously (Rydeen and Waxman, 2016). The embryos were kept in the drug solution until they were at 72 hpf and were analyzed for hemorrhages.

### qPCR

cDNA preparation and qPCR were performed as previously described (Craig et al., 2015). Briefly, embryos were frozen on dry ice and homogenized through a 22-gauge needle. RNA was extracted using an RNA-Aqueous 4RT-PCR kit (Life Technologies). cDNA was obtained using the Superscript Vilo (Invitrogen, 11754-050). qPCR was performed under standard conditions using Power up SYBR Green Master Mix (Applied Biosystems) in a StepOne PCR machine (Thermo Fisher Scientific). Three independent RNA samples were used for the experimental comparison and qPCR for each gene was performed in duplicate. Expression levels of *bip*, *hsp5* and *mmp9* were normalized to *ef1α.* Data were analyzed using 2^−ΔΔCT^ Livak Method and Student's *t*-test. The following pairs of primers were used for qPCR:

*bip*: 5′-AAGAGGCCGAAGAGAAGGAC-3′ and 5′-AGCAGCAGAGCCTCGAAATA-3′;

*hsp5*: 5′-CGAAGAAGCCAGATATCGATGA-3′ and 5′-ACGGCTCTTTTCCGTTGAAG-3′;

*mmp9*: 5′-CAAATCTGTGTTCGTGACGTTT-3′ and 5′-TCCGTCGAATGTCTTGTAGTTG-3′;

*ef1α*: 5′-TCACCCTGGGAGTGAAACAGC-3′ and 5′-ACTTGCAGGCGATGTGAGCAG-3′.

### Sequencing in the FIA study

WES was performed at the Center for Inherited Disease Research (Johns Hopkins University, Baltimore, MD, USA) in 45 individuals (35 affected, ten unaffected) from seven multiplex IA families participating in the FIA study (Farlow et al., 2015). Patient samples used in WES and targeted sequencing were collected with the informed consent according to an Institutional Review Board-approved protocol. Targeted sequencing for the *COL22A1* locus was subsequently performed in 456 unrelated, familial FIA study samples using multiplex PCR with primers specific to the exons within the *COL22A1* locus. The Agilent SureSelect XT Custom ELID kit was used to capture targeted regions. Paired-end sequencing was performed using the same protocol as for the WES project.

### Image manipulations

Image panels were assembled in Adobe Photoshop CS5. Non-linear gamma adjustments using Levels function were used for many panels to increase the contrast and clarity of the images. In all cases, similar adjustments between control and experimental (mutant) embryos were performed.

## Supplementary Material

Supplementary information
