# Supplementary material for: Collagen COL22A1 maintains vascular stability and mutations in COL22A1 are potentially associated with intracranial aneurysms
Source: Dis Model Mech. 2018 Dec 12;11(12):dmm033654. doi: 10.1242/dmm.033654 (PMC6307901; doi:10.1242/dmm.033654)
Supplement: Supplementary information [file dmm-11-033654-s1.pdf]

SUPPLEMENTAL TABLES AND FIGURES

Genotype	No hemorrhages	Hemorrhages (any)	Hemorrhages - head	Hemorrhages - eyes	Hemorrhages - trunk	Hemorrhages - mixed
Wild-type - Experiment 1	54	11	3	7	1	0
Wild-type - Experiment 2	28	3	3	0	0	0
Wild-type - Experiment 3	45	4	4	0	0	0
col22a1+/- Experiment 1	81	9	5	2	0	2
col22a1+/- Experiment 2	27	2	2	0	0	0
col22a1-/- Experiment 1	100	26	19	5	2	0
col22a1-/- Experiment 2	91	31	9	10	3	9
col22a1-/- Experiment 3	38	20	9	11	0	0
Wild-type - total	127	18	10	7	1	0
col22a1+/- total	108	11	7	2	0	2
col22a1-/- total	229	77	37	26	5	9

Table S1. Hemorrhage counts and their distribution in *col22a1* mutant and control embryos. The summary of this table is shown in Figure 4C.

Disease Models & Mechanisms • Supplementary information

Genomic location of a SNP	ExAC-NFE frequency	Prediction (PolyPhen-2)	Damage Score (PolyPhen-2)	Prediction (SIFT)	Damage score (SIFT)
Chr8: 139820025(C/A) **L**494**I**	1.5×10^{-5}	Probably Damaging	0.990	Tolerated	0.24
Chr8: 139736897(G/T) **E**736**D**	6.05×10^{-5}	Probably Damaging	0.992	Tolerated	0.3
Chr8: 139697452(C/T) **P**989**L**	0	Probably Damaging	0.999	Damaging	0.01
Chr8: 139638441(G/A) **G**1237**R**	0	Probably Damaging	1.000	Damaging	0
Chr8: 139631706(G/A) **G**1273**S**	0	Probably Damaging	1.000	Damaging	0.05
Chr8: 139609153(C/T) **L**1476**F**	0	Probably Damaging	0.999	Tolerated	0.1
Chr8: 139601677(C/A) **P**1567**H**	0	Probably Damaging	1.000	Damaging	0.03

Table S2. Genomic location of mutant variants in *COL22A1* and their allelic frequencies identified in the Exome Aggregation Consortium (ExAC) Non-Finnish Exomes (NFE) database. Genomic location is based on GRCh37 / hg19 genome assembly. The conserved SNPs as compared among zebrafish, mouse and human were tested on PolyPhen-2 (Prediction of Functional Effects of Human nSNPs) (Adzhubei et al., 2010) and SIFT (Sorting Intolerant From Tolerant) (Ng and Henikoff, 2003) to predict tolerated and damaging substitutions.

Disease Models & Mechanisms • Supplementary information

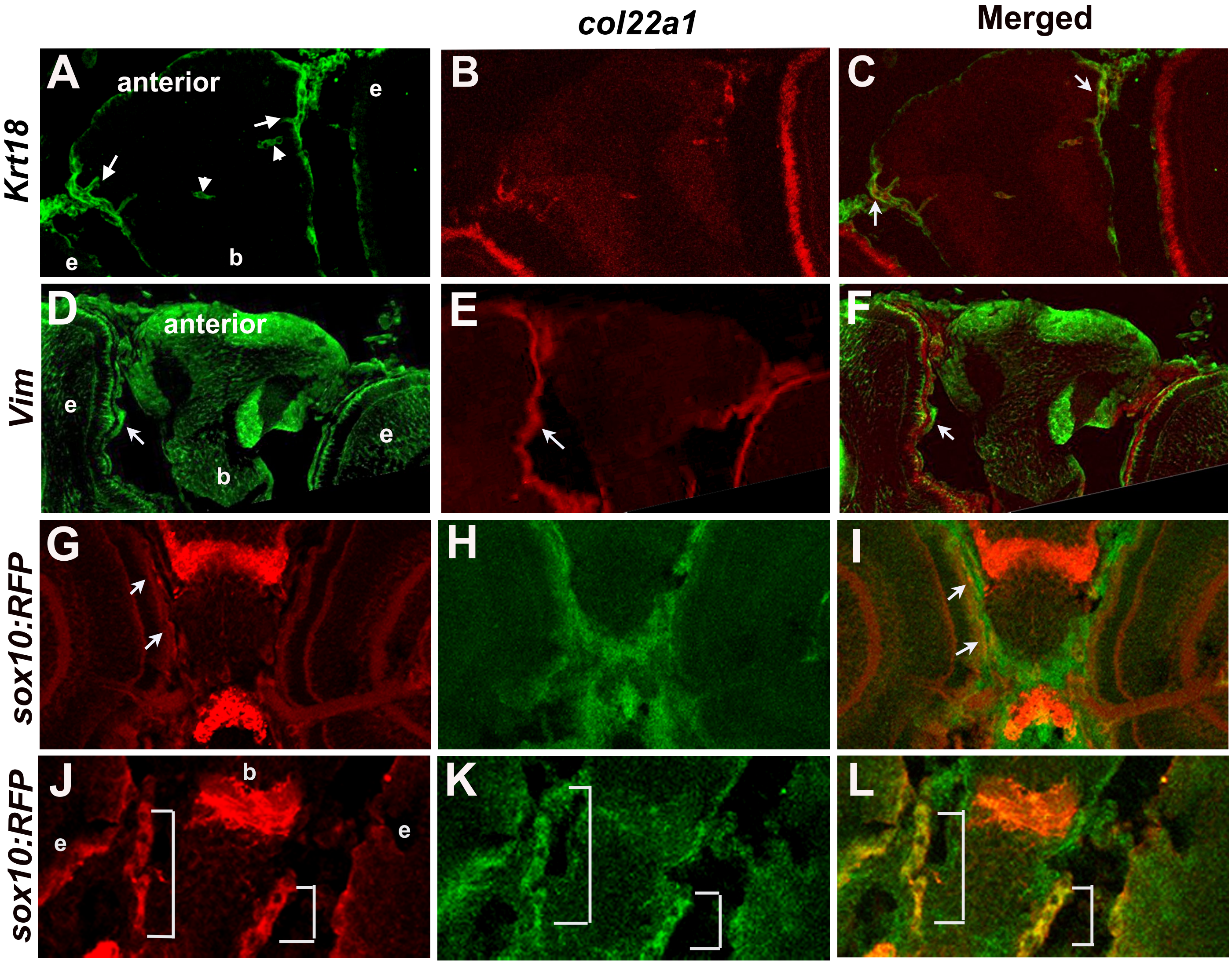

Figure S1. Longitudinal cranial sections of embryos stained by WISH/immunofluorescence for Keratin 18 (Krt18) (green) at 96 hpf (A-C) or Vimentin (Vim) (green) at 72 hpf (D-F), and for *col22a1* (green) and *sox10:RFP* (red), specific for neural crest derived cells at 72 hpf (G-L). Some fusiform (arrows) cells located in the meninges and cuboidal (arrowheads) stromal cells co-express Krt18 and *col22a1* (A-C). Many *col22a1* expressing cells are located adjacent to intermediate filament protein Vimentin (D-F). A subset of *col22a1*-positive cells adjacent to the eyes also are weakly positive for *sox10:RFP* (G-I). *Col22a1* and *sox10:RFP* co-expression was also detected in pharyngeal arches (J-L). b: brain; e: eye.

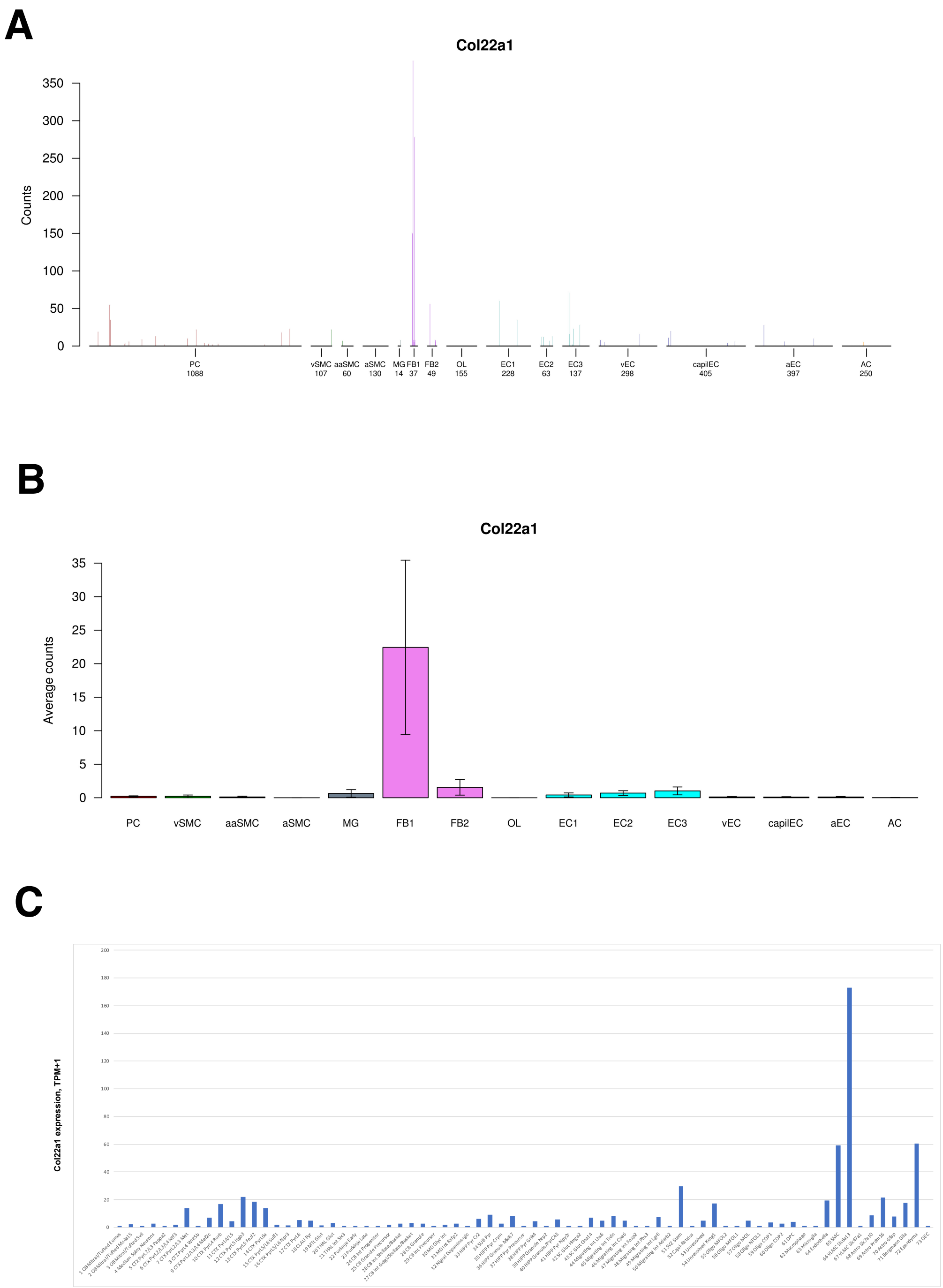

Figure S2. (A,B) Expression of *Col22a1* in each cell (A) and average *Col22a1* expression in each cluster (B) obtained from single cell profiling of adult mouse brain vascular cells (Vanlandewijck et al., 2018). Expression profile for *Col22a1* was obtained using the online database http://betsholtzlab.org/VascularSingleCells/database.html

Abbreviations: PC - Pericytes; SMC - Smooth muscle cells; MG - Microglia; FB - Vascular fibroblast-like cells; OL - Oligodendrocytes; EC - Endothelial cells; AC - Astrocytes; v - venous; capil - capillary; a - arterial; aa - arteriolar; 1,2,3- subtypes.

(C) Expression of *Col22a1* in different cell types from P2 and P11 mouse brain and spinal cord based on (Rosenberg et al., 2018). Expression is shown in TPM (transcripts per million) +1. Cell cluster types: 1 – 54 Different types of neurons; 55 – 61 oligodendrocytes; 62 – perivascular macrophage; 63 – microglia; 64 – vascular endothelial; 65 – smooth muscle cells; 66, 67 - vascular and leptomeningeal cells; 68 – 71 astrocytes; 72 – ependymal; 73 – olfactory ensheathing cells. Please see Table S3 in Rosenberg et al., 2018, for the detailed list of cell types and their markers.

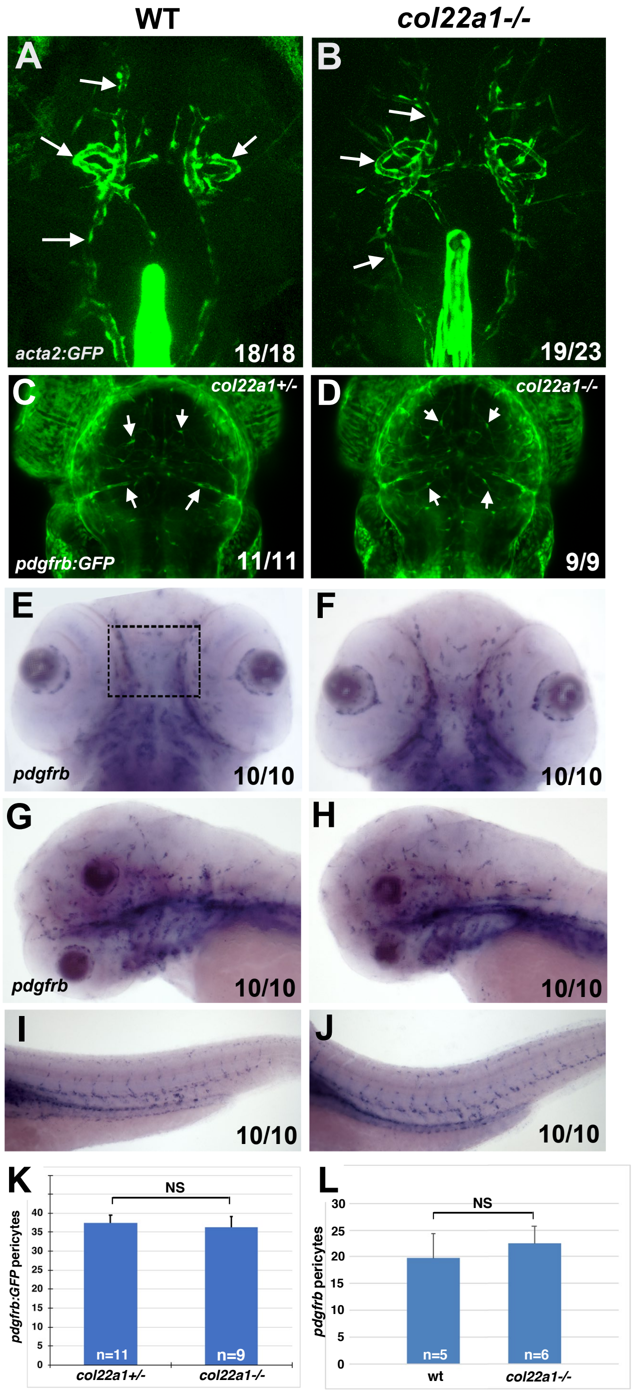

Figure S3. Mural cells are unaffected in *col22a1* mutants.
(A,B) Analysis of mural cell coverage in *acta2:GFP* wt and *col22a1-/-* embryos at 3 dpf (after heat stress starting at 1 dpf). There was no significant difference in GFP expression between the majority of *col22a1* and wild-type embryos. Confocal imaging of the cranial region; dorsal view; maximum intensity projection of selected slices is shown.
(C,D) Analysis of pericyte coverage in *TgBAC (pdgfrb:GFP) col22a1-/-* and sibling *col22a1+/-* embryos at 3 dpf (after heat stress starting at 1 dpf). No significant difference in pericyte number is apparent. Maximum intensity projection of selected stacks after confocal imaging is shown.
(E-J) Expression analysis of pericyte marker *pdgfrb* in wild-type (E,G,I) and *col22a1* mutants (F,H,J) by whole-mount in situ hybridization at 3 dpf. Ventral (E,F) and lateral (G,H) views of the head region; (I,J) lateral view of the trunk. No significant difference in pdgfrb expression between wild-type and mutant embryos was observed. (K) Quantification of *pdgfrb:GFP* cells in the cranial region. (L) Quantification of *pdgfrb* expressing cells in a selected region of cranial vasculature, shown approximately in a rectangle in (E). NS, not significant ($p>0.05$). Error bars show standard deviation in (K,L).

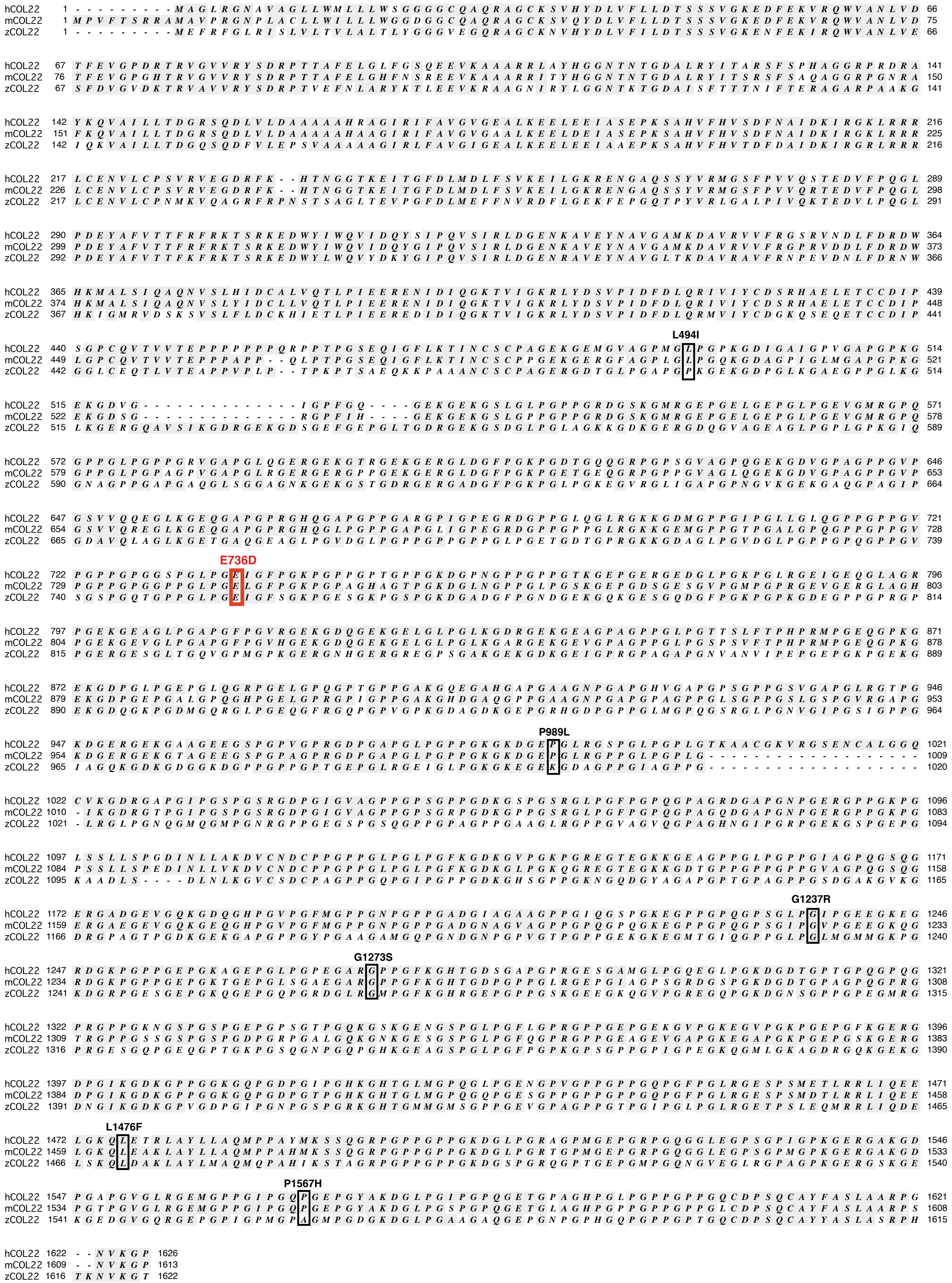

Figure S4. Conservation of COL22A1 SNP variants obtained from whole exome sequencing and targeted sequencing among humans (hCOL22), mice (mCOL22) and zebrafish (zCOL22). The conserved residues among the species are shaded in grey. SNPs identified by sequencing of affected individuals are boxed.

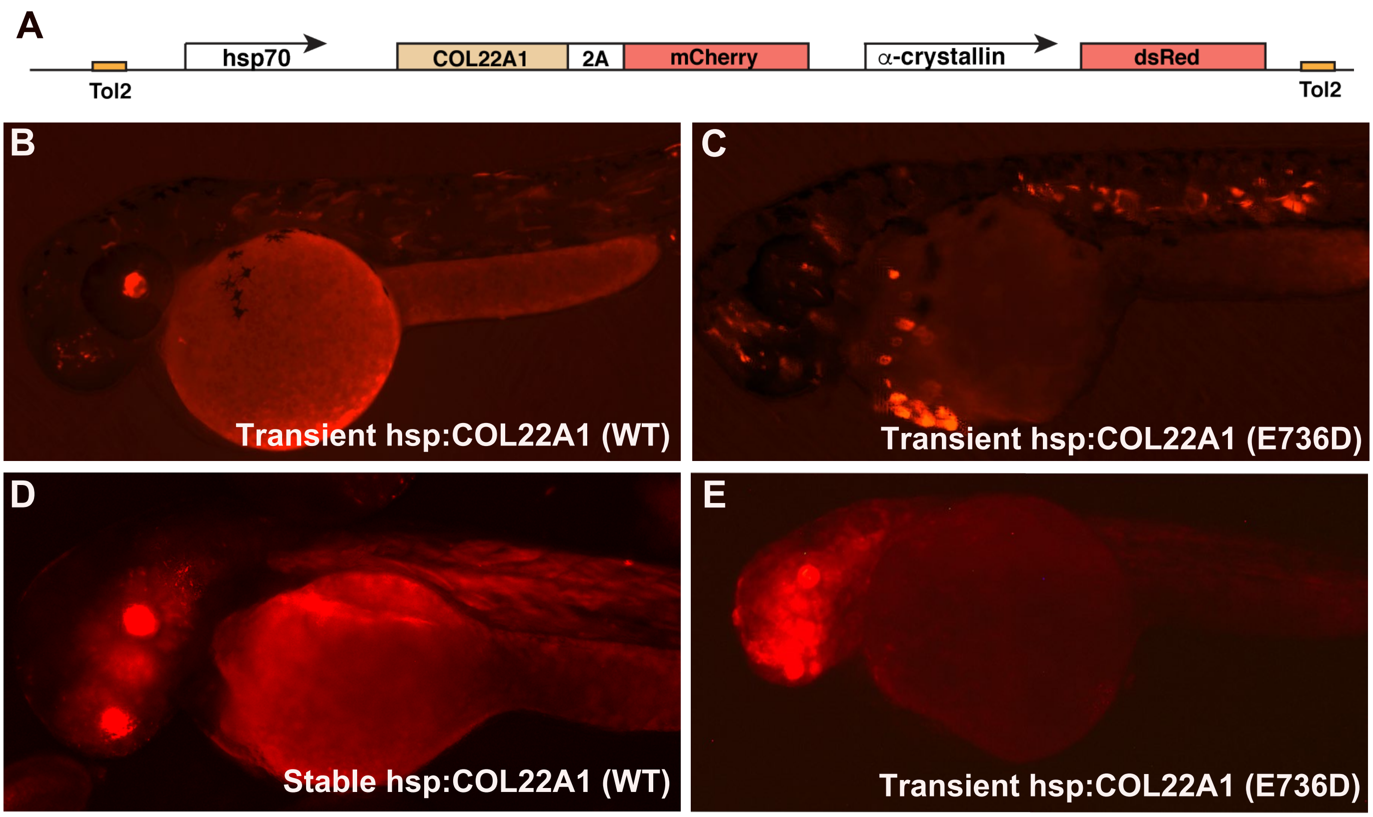

Figure S5. mCherry fluorescence in *hsp70:COL22A1-2A-mCherry* transgenic embryos. (A) A diagram of ***Tol2-hsp70:COL22A1-2A-mCherry;*** **α*-crystallin:dsRed-Tol2*** construct. The construct contains α*-crystallin* promoter driving dsRED expression in the lens for convenient screening to identify the transgenic embryos. (B,C) Transient expression in embryos injected with the heat shock inducible human WT and mutant E736D *COL22A1*. mCherry is expected to correlate with *COL22A1* expression. (D,E) Stable line established from the progeny of founders that were injected with the heat shock inducible constructs and were screened for mCherry expression.

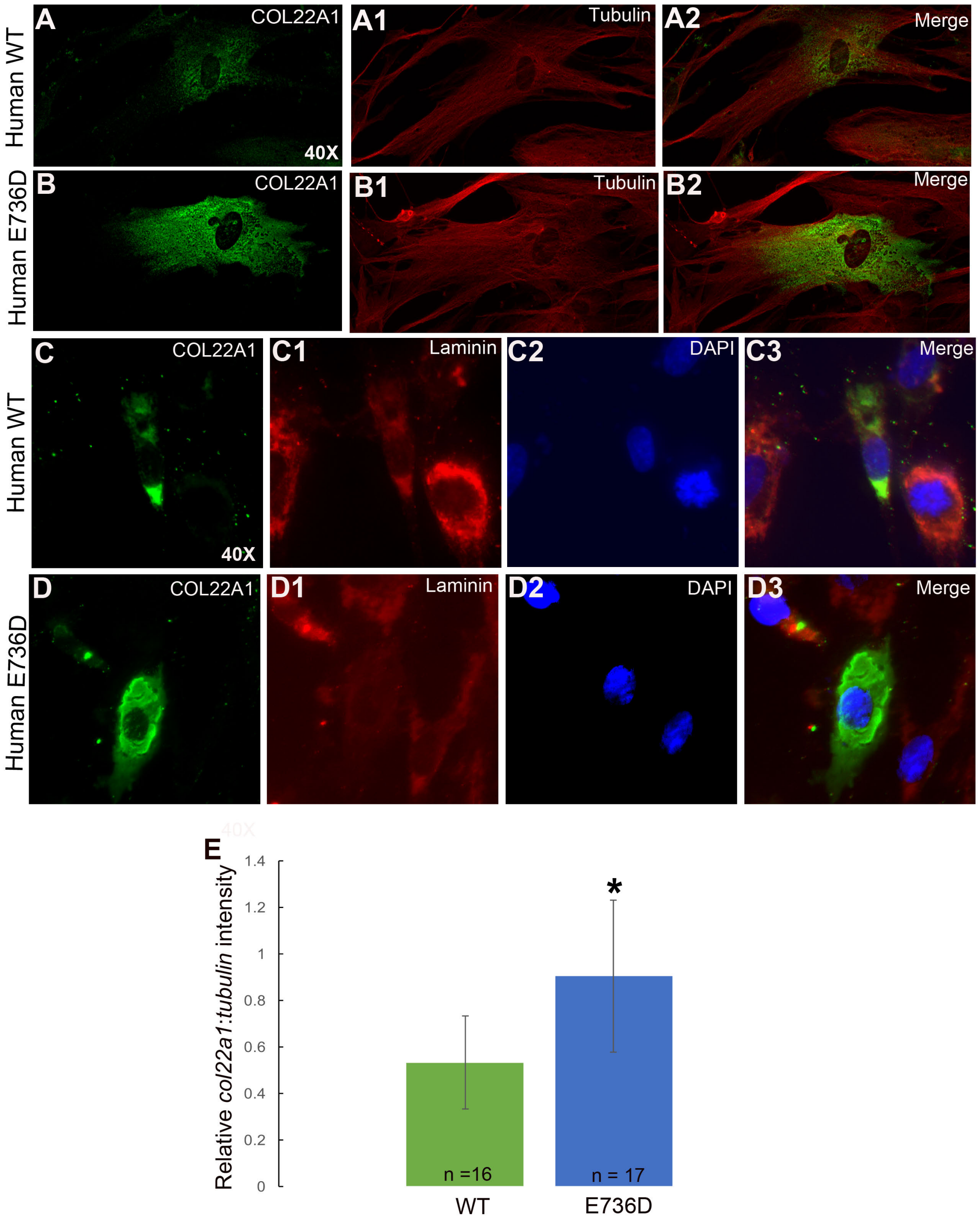

Figure S6. Human mutant E736D COL22A1 is retained in the cytoplasm of primary fibroblast cells. (A-B2) Confocal microscopy images show immunofluorescence using antibody against COL22A1 (green) and Tubulin (red). COL22A1 protein is retained in the cytoplasm of human E736D transiently transfected cells when compared with human WT COL22A1 transiently transfected cells according to the intensity of COL22A1 staining. (C-D3) Laminin expression (red) is reduced in E736D Col22A1 transfected cells. Immunofluorescence was analyzed by fluorescent compound microscopy. (E) Quantification of immunofluorescence intensity using Fiji software. The intensity of E736D COL22A1 staining is significantly higher in the cytoplasm than that of WT COL22A1. Error bars represent standard deviation, * $p < 0.05$, n: Number of cells used to calculate the intensity.

Supplemental Acknowledgements

FIA Study Investigators

Joseph Broderick, MD (University of Cincinnati, Study Principal Investigator, Executive committee, Steering Committee).
Daniel Woo, MD, MS (University of Cincinnati, Site Co-Investigator, Steering Committee)
Brett Kissela, MD, MS (University of Cincinnati, Site Co-Investigator)
Dawn Kleindorfer, MD (University of Cincinnati, Site Co-Investigator)
Alex Schneider, MD (University of Cincinnati, Site Co-Investigator)
Mario Zuccarello, MD (University of Cincinnati, Site Co-Investigator)
Andrew Ringer, MD (University of Cincinnati, Site Co-Investigator)
Ranjan Deka, PhD (University of Cincinnati, Genotyping Center Principal Investigator, Steering Committee, Executive Committee)
Tatiana Foroud, PhD (Indiana University, Co-Investigator Data Management/Linkage Analysis Center, Steering Committee, Executive Committee)
Robert D. Brown, Jr, MD, MPH (Mayo Clinic, Rochester, Co-Investigator Imaging Center, Steering Committee, Executive Committee)
John Huston, III, MD (Mayo Clinic, Rochester, Co-Investigator Imaging Center, Steering Committee, Executive Committee)
Irene Mesissner,MD (Mayo Clinic, Rochester, site Co-Investigator, Executive Committee)
David Wiebers, MD (Mayo Clinic, Rochester, site Co-Investigator, Executive Committee) Committee
Adnan I Qureshi, MD (University of Medicine and Dentistry of New Jersey, Site Investigator)
Peter A. Rasmussen,MD (Cleveland Clinic Foundation, Site Investigator)
E. Sander Connolly, Jr, MD (Columbia University, Site Investigator, Executive Committee)
Ralph L. Sacco, MD (Columbia University, Site Investigator)
Marc Malkaff, MD (University of Texas, Houston, Site Investigator)
Troy D. Payner, MD (Indianapolis Neurosurgical Group, Inc,/ Goodman Campbell Brain & Spine, Site Investigator)
Gary G. Ferguson, MD PhD (University of Western Ontario, London, Site Investigator)
E. Francois Aldrich, MD (University of Maryland, Baltimore, Site Investigator)
Guy Rouleau, MD, PhD (McGill University, University of Montreal, Notre Dame Hospital Site Investigator, Executive Committee)
Craig S Anderson, MD, PhD (Auckland UniServices, The George Institute, Sydney, Site Investigator, Executive Committee)
EdwardW. Mee, MD (Auckland Uniservices, Site Investigator)
Graeme J. Hankey, MD (Royal Perth Hospital, Site Investigator)
Neville Knuckey, MD (Sir Charles Gairdner Hospital, Site Investigator)
Peter L. Reilly, MD (Royal Adlaide Hospital, Site Investigator)
John D. Laidlaw, MD (Royal Melbourne Hospital, Site Investigator)
Paul D' Urso, MD (Alfred Hospital, Site Investigator)
Jeffrey V. Rosenfeld, MD (Alfred Hospital, Site Investigator)
Michael K Morgan, MD (Royal North Shore Hospital, Site Investigator)
Nicholas Dorsch, MD (Westmead Hospital, Site Investigator)
Michael Besser, MD (Royal Prince Alfred Hospital, Site Investigator)
H. Hunt Batjer, MD (Northwestern University, Site Investigator)
Michael T. Richard, MD (University of Ottawa, Site Investigator)
Amin Kassam, MD (University of Pittsburgh, Site Investigator)
Gary K. Steinberg, MD, PhD (Stanford University, Site Investigator)
S. Claiborne Johnston, MD, PhD (University of California, San Francisco, Site Investigator)
Nerissa U Ko, MD (University of California, San Francisco, Site Investigator, Steering Committee, Executive Committee)
Steven L. Giannotta, MD (University of Southern California, Site Investigator)

Disease Models & Mechanisms • Supplementary information

Neal F. Kassell, MD (University of Virginia, Site Investigator)
Bradford B. Worrall, MD (University of Virginia, Site Investigator, Steering Committee, Executive Committee)
Kenneth C. Lui, MD (University of Virginia, Site Investigator)
Aaron Dumont, MD (University of Virginia, Site Investigator)
David L. Tirschell, MD, MS (University of Washington, Seattle, Site Investigator)
Anthony M. Kaufmann, MD (University of Manitoba, Winnipeg, Site Investigator)
Winfield S. Fisher, III, MD (University of Alabama, Birmingham, Site Investigator)
Khaled Mohamed Abdel Aziz, Md, PhD (Allegheny General Hospital, Site Investigator)
Arthur L. Day, MD (Brigham and Women' s Hospital, Boston, Site Investigator)
Rose Du, MD, PhD (Brigham and Women' s Hospital Boston, Site Investigator)
Christopher Ogilvy, MD (Massachusetts General Hospital, Boston, Site Investigator)
Stephen B. Lewis, M.D. (University of Florida, Gainesville, Site Investigator)
Kieran P. Murphy, MD, FRCPC (Johns Hopkins University, Site Investigator)
Martin Radvany,MD (Johns Hopkins University, Site Investigator)
Dheerah Gandhi, MD (Johns Hopkins University, Site Investigator)
Lynda Lisabeth, PhD (University of Michigan, Ann Arbor, Site Investigator)
Aditya Pandey, MD (University of Michigan, Ann Arbor, Site Investigator)
Lewis Morgenstern, MD (University of Michigan, Ann Arbor, Site Investigator)
Colin Derdeyn, MD (Washington University in St. Louis, Site Investigator)
Carl Langefeld, PhD (Wake Forest School of Medicine, Consultant)
Joan Bailey-Wilson, PhD (Johns Hopkins University, Consultant)

Disease Models & Mechanisms • Supplementary information
